# Searching for new strategies against biofilm infections: Colistin-AMP combinations against *Pseudomonas aeruginosa* and *Staphylococcus aureus* single- and double-species biofilms

**DOI:** 10.1371/journal.pone.0174654

**Published:** 2017-03-29

**Authors:** Paula Jorge, Daria Grzywacz, Wojciech Kamysz, Anália Lourenço, Maria Olívia Pereira

**Affiliations:** 1 CEB - Centre of Biological Engineering, LIBRO - Laboratory of Research in Biofilms Rosário Oliveira, University of Minho, Braga, Portugal; 2 Lipopharm.pl, Zblewo, Poland; 3 Faculty of Pharmacy, Medical University of Gdansk, Gdansk, Poland; 4 ESEI - Escuela Superior de Ingeniería Informática, Universidad de Vigo, Ourense, Spain; 5 CEB - Centre of Biological Engineering, University of Minho, Braga, Portugal; Scripps Research Institute, UNITED STATES

## Abstract

Antimicrobial research is being pressured to look for more effective therapeutics for the ever-growing antibiotic-resistant infections, and antimicrobial peptides (AMP) and antimicrobial combinations are promising solutions. This work evaluates colistin-AMP combinations against two major pathogens, *Pseudomonas aeruginosa* and *Staphylococcus aureus*, encompassing non- and resistant strains. Colistin (CST) combined with the AMP temporin A (TEMP-A), citropin 1.1 (CIT-1.1) and tachyplesin I linear analogue (TP-I-L) was tested against planktonic, single- and double-species biofilm cultures. Overall synergy for planktonic *P*. *aeruginosa* and synergy/additiveness for planktonic *S*. *aureus* were observed. Biofilm growth prevention was achieved with synergy and additiveness. Pre-established 24 h-old biofilms were harder to eradicate, especially for *S*. *aureus* and double-species biofilms; still, some synergy and addictiveness was observed for higher concentrations, including for the biofilms of resistant strains. Different treatment times and growth media did not greatly influence AMP activity. CST revealed low toxicity compared with the other AMP but its combinations were toxic for high concentrations. Overall, combinations reduced effective AMP concentrations, mainly in prevention scenarios. Improvement of effectiveness and toxicity of therapeutic strategies will be further investigated.

## Introduction

Microbial adhesion onto surfaces with subsequent formation of biofilms is associated with 65–80% of human clinical infections [1]. Biofilms are complex networks of microorganisms protected by a self-produced polymeric matrix that gives them increased resistance towards antibiotherapy [2].

Biofilms of *P*. *aeruginosa*, an opportunistic Gram-negative pathogen, are notorious for causing chronic lung infections, e.g., in cystic fibrosis patients [3], and biomaterial-associated infections (e.g., catheters and contact lenses) [4]. Likewise, biofilms of the Gram-positive *S*. *aureus* are related to osteomyelitis, indwelling device infections, periodontitis, endocarditis and several chronic infections [5]. Often, these microorganisms co-occur in these biofilm-related infections, among others (e.g., diabetic foot ulcers and other wounds) [6]. Moreover, both bacteria are prominent examples of the ever-growing phenomenon of multi-drug resistance (MDR) strains [3,5].

Currently, the screening of alternative antimicrobial actions and the combination of agents are regarded as very promising towards the development of new, more effective antimicrobial approaches against biofilm-forming and MDR bacteria [7]. Antimicrobial peptides (AMP) are good candidates due to their alternative mechanisms of action, usually involving cell membrane damage, and general unspecific molecular targets, which reduces the chance of acquired resistance. Moreover, AMP can influence processes supporting antimicrobial action, such as cytokine release, chemotaxis, antigen presentation, angiogenesis and wound healing [8].

Strategies based on antimicrobial combinations seek to reduce individual agent concentrations and achieve synergic activity. These successful combinations can increase the antimicrobial spectrum while preventing the emergence of resistance and reducing toxicity and side effects. Synergy testing is encouraged in clinical treatments for MDR strains, namely *P*. *aeruginosa* associated pulmonary exacerbation [9]. Specifically, Colistin (CST), a well-known last resort AMP antibiotic, is often combined with other antibiotics for the treatment of MDR bacterial infections [10]. Moreover, previous reports showed that synergic combinations of CST with other AMP are able to eliminate CST toxicity in mammalian cells [11]. In fact, the premise that AMP could act in synergy with each other can be the reason for the wide array of peptides present in a single host, which reduces the necessary concentration of each AMP in order to effectively kill microorganisms [12].

Current studies are evaluating the potential AMP-related combinations, but most studies are combining AMP with traditional antibiotics. So far, only a few works tested AMP combinations and even less used biofilms as the microbial mode of growth [13].

Therefore, this work aims to contribute to this research by evaluating the prophylactic and therapeutic activity of the AMP citropin 1.1 (CIT-1.1), temporin A (TEMP-A) and a linear analogue of tachyplesin I (TP-I-L) in combination with CST. Novelty lays on the use of these specific Colistin-AMP combinations on *P*. *aeruginosa* and *S*. *aureus* single- and double-species biofilms formed by several strains, including MDR strains, along with the cytotoxicity evaluation of the most active combinations. To the best of our knowledge, there are no other works describing the effects of similar combinations for biofilms of *P*. *aeruginosa* and *S*. *aureus*.

## Materials and methods

### Peptides

CST sulfate salt (PubChem CID: 5311054) was purchased from Sigma in a white powder formula. A linear version of TP-I (PubChem CID: 16129721) without the two disulphide bridges was purchased from GenScriptThe AMP CIT-1.1 (PubChem CID: 10351598) and TEMP-A (PubChem CID: 9920205) were manually synthesised by a solid-phase synthesis method on polystyrene AM-RAM resin, using the Fmoc/tButyl strategy [14]. Coupling was performed with the HOBt/DIPCDI method and the Fmoc protecting group was removed with 20% piperidine. Crude peptides were cleaved from the resin using a mixture of trifluoroacetic acid (TFA), triisopropylsilane (TIS) and water as scavengers. The final products were purified by reverse-phase high performance liquid chromatography (RP-HPLC) in a mixture of acetonitrile-water with 0.1% TFA as the eluent. The molecular weight of the peptides was determined by MALDI-TOF. All peptides were solubilised in sterile distilled water and stored at -20°C as stock solutions of 1 mg mL^-1^.

### Bacterial strains and culture conditions

This work considered the reference strains *P*. *aeruginosa* PAO1 and *S*. *aureus* ATCC 25923 and the clinical isolates (CI): *P*. *aeruginosa* U147016-1 and *P*. *aeruginosa* I92198-1 (MDR), isolated, respectively, from an urinary infection and a central venous catheter at the S. Marcos Hospital in Braga, Portugal; *S*. *aureus* GB 2/1, isolated from explanted voice prostheses at the University Medical Center of Groningen, Netherlands; and *S*. *aureus* PD95.2 (MRSA), isolated from a peritoneal dialysis catheter from the Division of Nephrology of the Centro Hospitalar do Porto (Hospital Santo Antonio and Vila Nova Gaia/Espinho). All CI strains belong to the Centre of Biological Engineering’s collection of the University of Minho. Stocked cells at -70°C were streaked and grown overnight at 37°C on a tryptic soy agar (TSA) (Liofilchem) plate. Each strain was inoculated in 20 ml of Mueller Hinton broth (MHB) (Liofilchem) and 20 ml of tryptic soy broth (TSB) (Liofilchem), for planktonic and biofilm culturing, respectively. Cultures were incubated overnight (37°C, 120 rpm), centrifuged (9000 × *g*, room temperature, 5 min) and re-suspended in MHB (for planktonic growth) or TSB (for biofilm growth) until reaching 1x10^6^ CFU mL^-1^ or 2x10^6^ CFU mL^-1^ (by measuring absorbance at 640 nm), respectively.

### Planktonic susceptibility towards single AMP

The effect of AMP on planktonic cultures was determined by the minimum inhibitory concentration (MIC), following the Clinical and Laboratory Standards Institute (CLSI) method by broth microdilution [15], and the minimum bactericidal concentration (MBC). Briefly, in a round bottom 96-well polystyrene (PS) microtiter plate (Orange Scientific), 100 μL of the prepared bacterial suspensions were incubated (37°C, 120 rpm, 24 h) with 100 μL of serial dilutions of the AMP in MHB. The MIC values were established as the lowest concentrations capable of reducing by 99% the growth of the bacterial cells. Growth was determined by measuring absorbance at 640 nm. The MBC was determined by checking cell viability, after incubation, by CFU counting. The MBC values were established as the lowest concentrations capable of reducing over 99.9% of the number of cells.

### Planktonic susceptibility to AMP combinations

The susceptibility of planktonic cells to AMP combinations was assessed by the checkerboard microdilution assay. AMP were paired in serial two-fold increasing concentrations, until just below the MIC, and following the conditions aforementioned for the MIC and MBC assays. In order to evaluate potential synergy, the fractional inhibitory concentration index (FICI) (Eq 1) and the fractional bactericidal concentration index (FBCI) (Eq 2) were calculated by comparing the MIC and the MBC of each individual AMP (A_alone_ and B_alone_) against the concentrations achieved by the AMP combination (A_comb A/B_ and B_comb A/B_) [9].

$$\text{FICI }={\text{FIC}}_{\text{A}}+{\text{FIC}}_{\text{B}}=\frac{\text{MIC }({\text{A}}_{\text{comb A}/\text{B}})}{\text{MIC }({\text{A}}_{\text{alone}})}+\frac{\text{MIC }({\text{B}}_{\text{comb A}/\text{B}})}{\text{MIC }({\text{B}}_{\text{alone}})}$$ (1)

$$\text{FBCI }={\text{FBC}}_{\text{A}}+{\text{FBC}}_{\text{B}}=\frac{\text{MBC }({\text{A}}_{\text{comb A}/\text{B}})}{\text{MBC }({\text{A}}_{\text{alone}})}+\frac{\text{MBC }({\text{B}}_{\text{comb A}/\text{B}})}{\text{MBC }({\text{B}}_{\text{alone}})}$$ (2)

The interpretation of the breakpoint values for FICI and FBCI was as follows: ‘synergy (S)’ (FICI or FBCI ≤ 0.5), ‘additiveness (Ad)’ (0.5 < FICI or FBCI ≤ 1), ‘indifference (I)’ (1 < FICI or FBCI ≤ 4) and ‘antagonism (A)’ (FICI or FBCI > 4.0) [9].

### Inhibition of biofilm formation by AMP combinations

AMP combinations were tested for their ability to impair biofilm formation, as a prophylactic approach. Biofilms were developed according to the modified microtiter plate test proposed by Stepanović *et al*. [16]. Briefly, 100 μL of the prepared bacterial suspensions were transferred to a flat-bottom 96-well polystyrene microtiter plate (Orange Scientific), adding 100 μL of two-fold AMP solution diluted in TSB in the wells (or 50 μL + 50 μL of different four-fold AMP solutions for the combinations tests). Tests covered individual AMP and all possible combinations with concentrations ranging from below to above the MIC. The plates were incubated (37°C, 120 rpm, 24 h) (N-Biotek Shaker & Incubator NB-205Q) to promote biofilm formation. Afterwards, the content of each well was removed by plate inversion and the wells were washed twice with distilled sterile water to remove planktonic cells. The plates were then analysed in terms of number of viable biofilm-cells.

### Treatment of pre-established biofilms with AMP combinations

Mimicking a therapeutic approach, AMP combinations were tested on 24 h-old single and double-species biofilms grown as described previously. For the double-species biofilms, 100 μL of each bacterial solution (1x10^6^ CFU mL^-1^) was added to each well. After biofilm development, the content of each well was removed by plate inversion and the wells were washed twice with distilled sterile water to remove planktonic cells. Then, 200 μL of AMP solution diluted in TSB or PBS were added to each well. AMP were dissolved in TSB and in PBS in order to compare the action of the AMP in more (TSB) and less (PBS) rich and favourable biofilm forming media. AMP were tested singly and in combination. The plates were incubated aerobically (37°C, 120 rpm) for a low (2 h) and an intermediate (6 h) time course of antimicrobial treatment, prior to the analysis of biofilm-cell viability. The concentrations tested (32–128 mg L^-1^) defined the maximum treatment time of 6 h, since individual CST is able to eradicate *P*. *aeruginosa* PAO1 biofilms above this time period [17].

### Biofilm biomass quantification

Total biofilm biomass was quantified using the crystal violet (CV) staining method adapted from Stepanović et al. [16]. Briefly, biofilms were fixed with 200 μL of pure methanol (Valente e Ribeiro, Lda.–Portugal) for 15 min. The plates were emptied by plate inversion, air dried and the fixed biofilm was then stained for 5 min with 200 μL of CV (Merck Gram's CV solution, 100%) stain. Excess stain was washed between 3 to 5 times with 200 μL of water until all excess stain is removed. The plates were emptied by plate inversion, air dried and the stain bound to the adherent biofilm was re-suspended with 200 μL of 33% (V/V) glacial acetic acid (Fischer Scientific). The final solution was measured for its absorbance at 570 nm.

### Quantification of biofilm cells’ viability

To determine the number of viable cells, biofilms were detached by scrapping the bottom and sides of the wells. The resulting cellular suspensions were vortexed for 30 s, serially diluted in distilled sterile water, plated on TSA, and incubated at 37°C overnight. For the analysis of double-species biofilms, the dilutions were also plated onto pseudomonas isolation agar (PIA) and mannitol salt agar (MSA), in order to isolate and count *P*. *aeruginosa* and *S*. *aureus* CFU, respectively. The number of viable biofilm-cells was expressed as log (CFU cm^-2^), considering that the surface area of the well occupied by 200 μL and available for biofilm adherence is 1.53 cm^2^.

### Biofilm cell analysis by Live/Dead fluorescence microscopy

Double-species biofilms were grown in the previously described conditions with some modifications. Briefly, 1 mL/well of bacterial suspension was placed in a 24-well PS microtiter plate in which a PS coupon (surface area of approximately 1 cm^2^) was inserted in order to allow for the biofilm to develop on its surface. After incubation, the coupons were removed and washed 3 times by placing them under ultra-pure water. The coupons were then transferred to wells containing either PBS or PBS supplemented with an AMP combination (that produced the best treatment outcomes) and incubated for 6 h. Afterwards, coupons were washed again as described and briefly air-dried before adding 20 μL of a solution containing 50 μM SYTO^®^ BC green fluorescent nucleic acid stain (Thermo Fisher Scientific) and 1.5 mM Propidium Iodide (PI) (Thermo Fisher Scientific). These concentrations were previously optimized for double-species biofilms within our research group. Coupons were incubated for 10 min at room temperature and away from light and then observed in an Olympus BX51 fluorescence microscope. This experiment was performed with two coupon replicates.

### Effectiveness analysis of the AMP combinations on biofilms

The effectiveness of AMP combinations in both prophylactic and therapeutic approaches was assessed by comparing the action of the individual AMP with the action of the combinations. Two methodologies of data analysis were followed. Firstly, data was analysed strictly in terms of statistical differences, in which the conclusions were drawn as follows: ‘synergy (S)’–the combined action is superior to the sum of the individual actions; ‘additiveness (Ad)’–the combined action is equal to the sum of the individual actions; ‘indifference (I)’–the combined action is equal to the action of the most active peptide alone; ‘antagonism (A)’–the combined action is inferior to the action of the most active peptide alone. This analysis was performed for the biomass and cell viability data. Next, a more conservative approach was also followed in order to assess the biological significance of the previously drawn conclusions. Here, the action of the combinations was compared with the action of the most active individual AMP in terms of cell viability and conclusions were drawn as: ‘synergy (S)’–≥ 2 log decrease; ‘additiveness (Ad)’– 1 ≤ log < 2 decrease; ‘indifference (I)’–< 1 log decrease; ‘antagonism (A)’–≥ 2 log increase [18].

### Cytotoxicity evaluation

Fibroblasts BALB/3T3 (ATCC CCL-163) were used to evaluate the toxicity of the individual and combined AMP in mammalian cells. Cells were cultured in Dulbecco's Modified Eagle's Medium (DMEM) supplemented with 10% of fetal bovine serum (FBS) and 1% penicillin/streptomycin at 37°C with 5% CO_2_. After achieving a minimum of 80% confluence, cells were detached using trypsin and diluted with supplemented DMEM to a density of 1×10^5^ cells mL^-1^. Then, 300 μL of cell suspension were added to a flat-bottom 48-well polystyrene microtiter plate (Orange Scientific) and the plates were incubated at 37°C with 5% CO_2_. After, the medium was removed, the cells were washed with PBS and 300 μL of AMP solutions, previously diluted in supplemented DMEM, were added. The plates were incubated for another 24 h. Afterwards, the medium was removed, the cells were washed with PBS and a mixture of MTS [3-(4,5-carboxymethoxyphenyl)-2-(4-sulfophenyl)-2H-tetrazolium] (Promega) and DMEM without phenol at 9% (V/V) was added to each well. After 1 h of incubation at 37°C away from light, the absorbance was measured at 490 nm and the results were expressed as percentage of viable cells (%) compared to the positive control.

### Statistical analysis

All tests were performed with at least two reproductions with no less than two replicates each. The examination of the activity of AMP combinations in biofilms encompassed the analysis of variance (ANOVA) followed by the Bonferroni's correction method in multiple comparison test. All statistical evaluations were carried out in GraphPad Prism 5.03 (GraphPad Software Inc., San Diego, CA). Heatmaps for the checkerboard assays were constructed using Plotly [19].

## Results

### Planktonic susceptibility to single AMP

CST showed the best bacteriostatic and bactericidal activities against *P*. *aeruginosa* with MIC and MBC values equal to 2 mg L^-1^, except for the CI MDR strain for which the MBC was 4 mg L^-1^ (Table 1), indicating sensitivity of these strains to this AMP. CST was followed by TP-I-L (MIC and MBC values equal to 8 mg L^-1^ for the PAO1 and CI strains and 16 and 32 mg L^-1^, respectively, for the CI MDR strain). In contrast, CST was the least effective AMP in *S*. *aureus* (MIC and MBC values between 32 and > 128 mg L^-1^). TP-I-L was the AMP that showed the best anti-staphylococcal effect (MIC and MBC values range of 4–8 mg L^-1^), with the exception of the CI MDR strain (MIC and MBC values of 8 and 32 mg L^-1^, respectively), for which the MIC of TEMP-A was slightly (4 mg L^-1^). TEMP-A and CIT-1.1 were more effective in *S*. *aureus* than in *P*. *aeruginosa* (MIC and MBC values range of 4–32 mg L^-1^ *vs* ≥ 128 mg L^-1^). In general, the MBC values were equal or higher than the MIC values and little difference was found between the susceptibilities of the reference strains and the CI. On the other hand, the CI MDR and CI MRSA strains were slightly less susceptible than the other strains, notably in terms of MBC values.

**Table 1:** Antibacterial activity of CST, TEMP-A, CIT-1.1 and TP-I-L against planktonic *P*. *aeruginosa* and *S*. *aureus*.

Strain		CST		TEMP-A		CIT-1.1		TP-I-L	
		MIC	MBC	MIC	MBC	MIC	MBC	MIC	MBC
***P*. *aeruginosa***	**PAO1**	2	2	>128	>128	128	128	8	8
	**CI**	2	2	>128	>128	128	>128	8	8
	**CI MDR**	2	4	>128	>128	128	>128	16	32
***S*. *aureus***	**ATCC 25923**	64	>128	8	16	16	16	4	8
	**CI**	32	128	4	8	8	8	4	4
	**CI MRSA**	32	>128	4	32	8	32	8	32

MIC and MBC values are in mg L^-1^.

### Planktonic susceptibility to AMP combinations

The best outcomes for the AMP combinations against planktonic bacteria are shown in Table 2 (see S1 and S2 Files for detailed outcomes). The FICI and the FBCI values show that most of the combinations have synergic activities against *P*. *aeruginosa* strains, but the CI MDR strain was slightly less susceptible to the action of the combinations. Regarding *S*. *aureus*, the susceptibility profiles of the reference and the CI strains were similar. Overall, *S*. *aureus* was less susceptible than *P*. *aeruginosa* to the combinations, and only the combination of CST with TP-I-L showed synergic outcomes. CST with TEMP-A and CST with CIT-1.1 combinations resulted in FICI and FBCI values below 0.3 in *P*. *aeruginosa* PAO1, which indicates a substantial increase in the antimicrobial effectiveness of these peptides when combined. The FBCI of some of the combinations for the CI MDR and CI MRSA strains were not detected in the range of concentrations tested. This might be explained by the lower bactericidal susceptibility demonstrated by these strains in the susceptibility assays using the isolated AMP (Table 1).

**Table 2:** Best outcomes for the combinational activities of CST with TEMP-A, CIT-1.1 and TP-I-L against planktonic *P*. *aeruginosa* and *S*. *aureus*.

Strain		CST +					
		TEMP-A		CIT-1.1		TP-I-L	
		FICI	FBCI	FICI	FBCI	FICI	FBCI
***P*. *aeruginosa***	**PAO1**	**S**	**S**	**S**	**S**	**S**	**S**
		0.27	0.27	0.26	0.25	0.38	0.50
		0.5 + 4	0.5 + 4	0.5 + 1	0.5 + 16	0.25 + 2	0.5 + 2
	**CI**	**S**	**S**	**S**	**S**	**S**	**Ad**
		0.50	0.50	0.38	0.38	0.50	0.75
		1 + 1	1 + 1	0.25 + 32	0.25 + 32	0.5 + 2	0.5 + 4
	**CI MDR**	**Ad**	**S**	**Ad**	**ND**	**Ad**	**Ad**
		0.75	0.38	0.75		0.63	0.63
		0.5 + 64	0.5 + 64	0.5 + 64		1 + 2	0.5 + 16
***S*. *aureus***	**ATCC 25923**	**Ad**	**Ad**	I	**Ad**	**S**	**S**
		0.56	0.75	1.03	0.56	0.50	0.25
		2 + 4	16 + 2	1 + 16	32 + 1	16 + 1	16 + 1
	**CI**	**Ad**	**Ad**	**Ad**	I	**Ad**	**S**
		0.75	0.51	1.00	1.02	0.75	0.50
		8 + 2	1 + 4	16 + 4	1 + 8	8 + 1	1 + 2
	**CI MRSA**	**Ad**	**ND**	**Ad**	**ND**	**Ad**	**S**
		0.63		0.75		0.63	0.19
		4 + 2		16 + 2		4 + 4	16 + 4

Data is shown per row: 1^st^—outcome; 2^nd^—lowest values of FICI and FBCI; 3^rd^—CST + AMP concentrations. The more positive outcomes (S and Ad) are shown in bold. ND—not detected in the range of concentrations tested.

Overall, similar outcomes for other strains of *P*. *aeruginosa* and *S*. *aureus* were obtained in previous reports [20,21]. No antagonistic interactions were observed.

The outcomes on Table 2 were achieved using lower concentration values than the ones determined for the MIC and MBC of all AMP for both bacteria. Most of the non-synergic outcomes were considered additive, with the highest value of FICI or FBCI equal to 1, which means that the MIC or MBC were decreased up to two-fold. These specific combinations are non-synergistic but helpful since they still allow for the use of lower concentrations, which are related with lower development of resistance, secondary effects and toxicity [9].

### Inhibition of biofilm formation by AMP combinations

The anti-biofilm activity of CST combined with other AMP was also investigated in the prevention of biofilm formation by analysing the biofilm cells’ viability (Table 3) and by quantifying biofilm biomass (data not shown). Table 3 shows the best outcomes achieved (see S1 and S2 Figs for detailed outcomes). Based on statistical differences, most of the AMP combinations produced synergic outcomes (61%) with high log reductions ranging from 3.3 to 7.7 (Table 3), often with almost or total absence of biofilm-cell viability (data not shown). However, outcomes of reference and CI strains showed some differences. For example, in *S*. *aureus*, CST combinations produced opposite results: CST with CIT-1.1 resulted in antagonistic and synergic outcomes for the reference and CI strains, respectively; and CST with TEMP-A resulted in synergic outcomes for the reference and CI MRSA strains in contrast to the indifference manifested by the CI strain. Statistical analysis of the biofilm biomass data deemed all combination outcomes as indifferent, which might be explained by two factors: i) the decrease in cell viability was not accompanied by a decrease in biomass, which is plausible since AMP action does not target the biofilms matrix, thus making biomass quantification a poor indicator of AMP action; ii) the absorbance values for some of the strains were close to the lower detection limit, which limited the comparison between the different conditions tested. Hence, it is plausible to assume that for the present strains, the biofilm biomass is not a good basis for the statistical comparison of combination outcomes.

**Table 3:** Best outcomes for the combinational activities of CST with TEMP-A, CIT-1.1 and TP-I-L in inhibiting the biofilm growth of *P*. *aeruginosa* and *S*. *aureus*.

Strain		CST +		
		TEMP-A	CIT-1.1	TP-I-L
***P*. *aeruginosa***	**PAO1**	**Ad** / **Ad**	**S** / **S**	**Ad** / I
		7.0	7.7	7.3
		8 + 16	4 + 8	8 + 16
	**CI**	**S** / **S**	**S** / **S**	**S** / **S**
		5.7	6.3	6.1
		8 + 8	4 + 16	4 + 8
	**CI MDR**	**S** / **Ad**	I / I	**Ad** / **Ad**
		4.9	3.2	6.2
		8 + 16	8 + 8	4 + 8
***S*. *aureus***	**ATCC 25923**	**S** / **S**	A / I	**S** / I
		4.8	1.7	3.7
		32 + 64	16 + 32	32 + 64
	**CI**	I / I	**S** / **Ad**	**S** / **Ad**
		3.5	3.3	5.8
		16 + 32	16 + 32	16 + 64
	**CI MRSA**	**S** / **Ad**	**Ad** / **Ad**	**S** / **S**
		4.9	2.9	4.6
		16 + 64	32 + 64	32 + 32

Data is shown per row: 1^st^—outcome shown as their statistical / biological significance; 2^nd^—average log (CFU cm^-2^) reduction achieved compared to the positive control; 3^rd^—CST + AMP concentrations. The more positive outcomes (S and Ad) are shown in bold.

In terms of biological significance, the three combinations were considered synergic for the *P*. *aeruginosa* CI and the combination of CST with CIT-1.1 was synergic for the reference and CI strains of this bacterium. None of the combinations were considered synergic for the CI MDR strain. In *S*. *aureus*, the combinations of CST with TEMP-A and with TP-I-L were synergic for the reference and CI MRSA strains, respectively, and the other two CST combinations were considered additive for the CI and CI MRSA strains. The remaining outcomes were considered indifferent.

It was interesting to observe the relevance of AMP concentrations in combination results, with a tendency of improvement (more synergic outcomes) when using higher AMP concentrations (data not shown). A possible explanation is the fact that AMP need to reach a threshold concentration in order to enter and cross the lipid bilayer and hence exert their antimicrobial function [22].

### Treatment of pre-established single-species biofilms with AMP combinations

**Fig 1:**
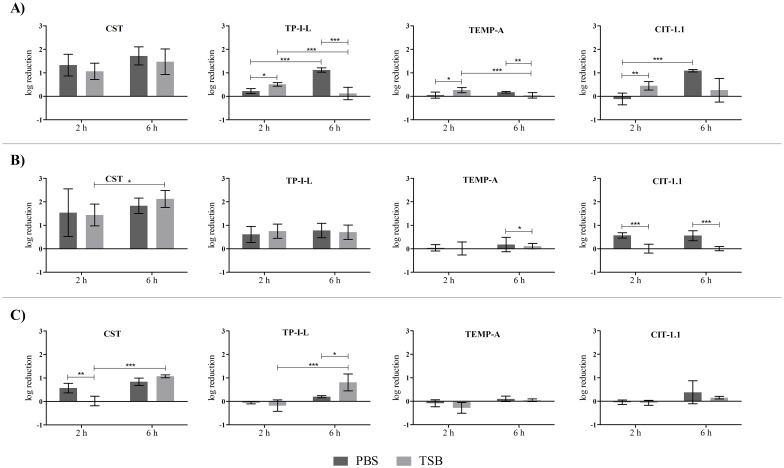
Treatment media and treatment time influence on single AMP activity on 24 h-old *P*. *aeruginosa* biofilms. Concentrations used were 32 mg L^-1^ of CST and 64 mg L^-1^ of the other AMP. A) *P*. *aeruginosa* PAO1, B) *P*. *aeruginosa* CI and C) *P*. *aeruginosa* CI MDR.

**Fig 2:**
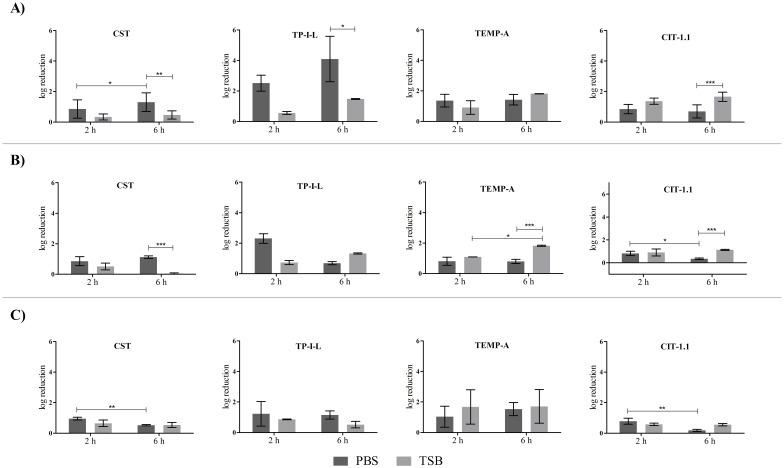
Treatment media and treatment time influence on single AMP activity on 24 h-old *S*. *aureus* biofilms. Concentrations used were 64 mg L^-1^ of CST and 32 mg L^-1^ of the other AMP. A) *S*. *aureus* ATCC 25923, B) *S*. *aureus* CI and C) *S*. *aureus* CI MRSA.

**Fig 3:**
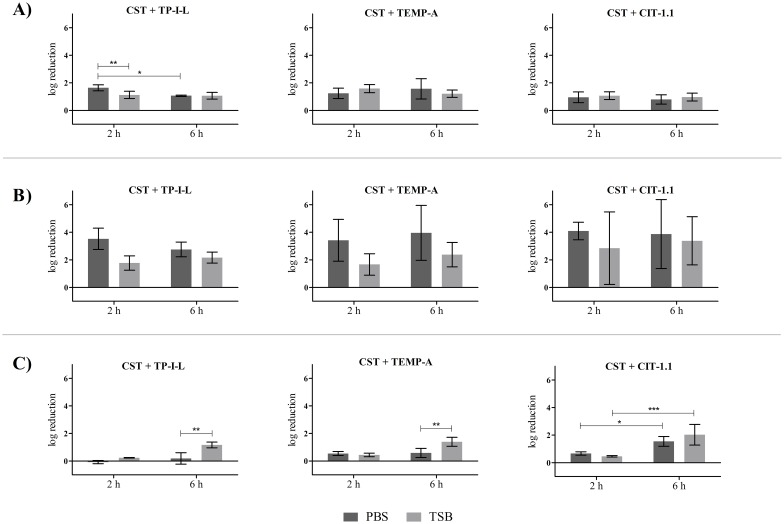
Treatment media and treatment time influence on AMP combination activity on 24 h-old *P*. *aeruginosa* biofilms. Concentrations used were 32 mg L^-1^ of CST and 64 mg L^-1^ of the other AMP. A) *P*. *aeruginosa* PAO1, B) *P*. *aeruginosa* CI and C) *P*. *aeruginosa* CI MDR.

**Fig 4:**
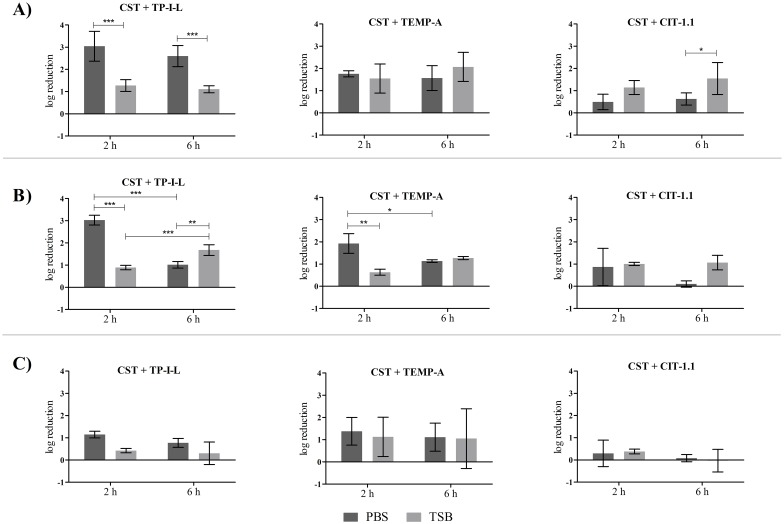
Treatment media and treatment time influence on AMP combination activity on 24 h-old *S*. *aureus* biofilms. Concentrations used were 64 mg L^-1^ of CST and 32 mg L^-1^ of the other AMP. A) *S*. *aureus* ATCC 25923, B) *S*. *aureus* CI and C) *S*. *aureus* CI MRSA.

The comparison between time course of antimicrobial treatment and growth medium while using individual AMP is depicted in Figs 1 and 2 for *P*. *aeruginosa* and *S*. *aureus*, respectively. Figs 3 and 4 depict a similar analysis for AMP combinations in *P*. *aeruginosa* and *S*. *aureus*, respectively.

CST was the most active AMP against *P*. *aeruginosa* biofilms, with some experiments reaching almost 3 log reduction (Fig 1B). In terms of anti-biofilm activity, CST was followed in descending order by TP-I-L, CIT-1.1 and TEMP-A, for all three strains (Fig 1). Some statistical differences were found between the two times of treatment and the two treatment media (*p* < 0.05). The most noticed differences relate to the two times of treatment for the PBS treated biofilms and the two media for both times of treatment. However, these differences were both associated with increases and decreases in log reductions, with no apparent correlation with the AMP used.

The most active AMP against *S*. *aureus* biofilms was TP-I-L, followed by CIT-1.1 and TEMP-A, and finally CST. TP-I-L was able to reduce the 24 h-old biofilm of *S*. *aureus* ATCC 25923 up to 6 log (total eradication) in 6 h (Fig 2A). Statistical differences were observed when comparing the two media at 6 h of time of treatment and also between the two times of treatment for both media (Fig 2). CIT-1.1 and TEMP-A caused a higher log reduction in TSB than in PBS at 6 h treatment time (*p* < 0.05) in two strains (ATCC 25923 and CI) and one strain (CI), respectively. On the other hand, TP-I-L and CST caused a higher log reduction in PBS than in TSB at 6 h treatment time (*p* < 0.05) in one strain (ATCC 25923) and two strains (ATCC 25923 and CI), respectively. Differences between the two treatment times were majorly seen for PBS, with a decrease in log reduction from 2 h to 6 h treatment time in three out of four cases.

Concerning the AMP combinations, a few statistical differences (*p* > 0.05) between media and treatment time for *P*. *aeruginosa* were observed, as is the case of the CI MDR strain, in which the combination CST + TP-I-L and CST + TEMP-A caused a higher log reduction in TSB than in PBS for 6 h treatment time (*p* < 0.05) (Fig 3C). Overall, the combinations were more effective against CI and CI MDR strains and, in some experiments, were able to reduce the 24 h-old biofilm up to 6 log (total eradication) in both 2 h and 6 h (Fig 3B and 3C).

In the case of *S*. *aureus*, some statistical differences between media and treatment time were observed, mainly for the combination of CST + TP-I-L, in which treatment with TSB caused overall lower log reductions compared with treatment with PBS (*p* < 0.05) (Fig 4). The combinations were slightly more effective against the reference strain (Fig 4A). Also, the combinations of CST + TP-I-L and CST + TEMP-A were generally more effective than the combination of CST + CIT-1.1.

Overall, neither the medium nor the time of treatment had an influence on the individual and combined AMP activity against *P*. *aeruginosa* and *S*. *aureus* biofilms in a way that could be clearly correlated with the AMP tested. Therefore, the effectiveness analysis of the combinations was performed for all the conditions tested.

Table 4 summarizes the best outcomes regarding the activity of the AMP combinations against pre-established single-species biofilms, using the following AMP concentrations: 32 mg L^-1^ of CST with 64 mg L^-1^ of the other AMP for *P*. *aeruginosa* and 64 mg L^-1^ of CST with 32 mg L^-1^ of the other AMP for *S*. *aureus*. Most of the results were of indifference for both bacteria, meaning that the action of the combinations was overall equal to the action of the most active single agent. Nevertheless, there were several positive outcomes, mainly for *P*. *aeruginosa* biofilms. For example, in *P*. *aeruginosa* CI biofilms, 7 out of a total of 12 outcomes were of synergy or additiveness, with reductions between 2.8 log and 3.9 log. Also, some synergic and additive outcomes were observed for the CI MDR strain when applying the combinations of CST with TEMP-A and CIT-1.1. Interestingly, synergic outcomes were more frequent for the CI strain when grown in PBS rather than TSB whereas the opposite was true for the CI MDR strain. Additionally, no substantial differences was observed concerning the two times of treatment. Regarding *S*. *aureus* biofilms, most of the outcomes were of indifference with some cases of additiveness. A comprehensive look of data gathered in Table 4 shows that there is at least one combination that achieved positive results for the reference and CI strains: CST + TEMP-A (PBS, 2 h). Finally, and as previously explained, the statistical analysis of the biofilm biomass data estimated all combination outcomes as indifferent.

**Table 4:** Best outcomes for the combinational activities of CST with TEMP-A, CIT-1.1 and TP-I-L in eradicating a 24 h-old single-species biofilm of *P*. *aeruginosa* and *S*. *aureus*.

Strain		CST +											
		TEMP-A				CIT-1.1				TP-I-L			
		2 h		6 h		2 h		6 h		2 h		6 h	
		PBS	TSB	PBS	TSB	PBS	TSB	PBS	TSB	PBS	TSB	PBS	TSB
***P*. *aeruginosa***	**PAO1**	**S** / I	**S** / I	I / I	A / I	A / I	I / I	I / I	I / I	A / I	I / I	**S** / I	I / I
		1.1	0.5	1.0	1.0	1.0	0.6	0.6	0.5	1.0	0.8	**2.1**	0.7
	**CI**	**S** / **Ad**	I / I	**S** / **S**	**Ad** / I	**S** / I	**S** / **Ad**	**S** / **Ad**	**S** / **Ad**	**S** / **Ad**	A / I	**S** / **Ad**	I / I
		**3.4**	1.8	**3.2**	**2.3**	**2.9**	**3.6**	**3.9**	**3.9**	**3.5**	1.5	**2.8**	**2.2**
	**CI MDR**	I / I	**S** / I	I / I	**S / Ad**	I / I	**S** / I	**S** / I	**S / Ad**	I / I	**Ad** / I	A / I	I / I
		0.2	0.4	0.6	1.4	0.4	0.5	1.5	**2.0**	0.0	0.2	0.2	1.2
***S*. *aureus***	**ATCC 25923**	**Ad** / I	**S** / I	I / I	A / I	I / I	I / I	A / I	**Ad** / I	I / I	**Ad** / I	I / I	I / I
		1.7	1.7	**2.7**	1.4	0.5	1.3	0.6	1.8	**2.7**	**2.3**	**3.6**	1.3
	**CI**	**S** / **S**	A / I	I / I	A / I	I / I	I / I	A / I	I / I	I / I	**S** / I	I / I	**S** / I
		**2.5**	0.5	1.1	1.2	0.9	1.0	0.1	1.0	1.9	0.9	**2.9**	1.9
	**CI MRSA**	I / I	A / I	I / I	I / I	I / I	I / I	I / I	I / I	I / I	A / I	I / I	I / I
		1.4	1.1	1.1	1.1	0.3	0.4	0.1	0.0	1.1	0.4	0.8	0.3

Data is shown per row: 1^st^—outcome shown as their statistical / biological significance; 2^nd^—average log (CFU cm^-2^) reduction achieved compared to the positive control. The more positive outcomes (S, Ad and ≥ 2 log reduction) are shown in bold.

### Treatment of pre-established double-species biofilms with AMP combinations

The effectiveness of the AMP combinations was also tested on 24 h-old double-species biofilms of *P*. *aeruginosa* and *S*. *aureus*. The treatment media chosen was PBS given it delivered the best results in the single-species biofilms. The best outcomes are presented in Table 5 (see S3–S5 Figs for detailed outcomes).

**Table 5:** Best outcomes for the combinational activities of CST with TP-I-L, TEMP-A and CIT-1.1 in eradicating 24 h-old double-species biofilms of *P*. *aeruginosa* PAO1 + *S*. *aureus* ATCC 25923, *P*. *aeruginosa* CI + *S*. *aureus* CI and *P*. *aeruginosa* CI MDR + *S*. *aureus* CI MRSA.

CFU Growth Media		TSA		PIA		MSA	
Treatment time		2 h	6 h	2 h	6 h	2 h	6 h
**CST +**	***P*. *aeruginosa* PAO1 + *S*. *aureus* ATCC 25923**						
	**TP-I-L**	**S** / **Ad**	**S** / **Ad**	**Ad** / **Ad**	**S** / **S**	I / **Ad**	I / I
		**3.8**	**3.4**	**3.6**	**4.6**	1.6	2.1
		64	128	64	128	32	128
	**TEMP-A**	I / I	**Ad** / I	I / I	**Ad** / I	I / I	**Ad** / I
		0.9	0.9	0.2	0.3	0.7	0.7
		64	16	64	128	128	16
	**CIT-1.1**	A / I	**Ad** / I	A / I	**Ad** / I	A / I	A / I
		0.4	1.4	0.2	0.5	0.5	0.5
		16	128	64	128	64	32
	***P*. *aeruginosa* CI + *S*. *aureus* CI**						
	**TP-I-L**	I / **Ad**	**Ad** / **Ad**	**Ad** / **Ad**	I / I	I / I	I / I
		**4.8**	**3.8**	**4.3**	**3.1**	**2.2**	**2.8**
		128	128	128	128	128	128
	**TEMP-A**	I / I	A / I	A / I	I / I	**Ad** / **Ad**	I / **Ad**
		1.6	**2.1**	1.8	**2.5**	1.2	1.5
		16	128	128	128	16	32
	**CIT-1.1**	I / I	I / I	I / I	I / I	**Ad** / **Ad**	**Ad** / **Ad**
		**2.0**	**2.7**	1.9	**2.3**	1.2	**2.2**
		128	128	128	128	32	16
	***P*. *aeruginosa* CI MDR + *S*. *aureus* CI MRSA**						
	**TP-I-L**	I / I	**S** / I	**Ad** / **Ad**	**Ad** / **Ad**	**S** / **S**	**Ad** / **Ad**
		0.7	1.0	1.5	1.3	**4.5**	**3.2**
		128	16	128	128	128	128
	**TEMP-A**	**Ad** / **Ad**	**Ad** / **Ad**	**Ad** / **Ad**	**S** / **Ad**	**S** / I	**S** / **Ad**
		0.7	1.3	1.1	1.8	0.4	1.8
		16	128	128	128	32	64
	**CIT-1.1**	**Ad** / I	**S** / **Ad**	**Ad** / I	**S** / **S**	I / I	**S / S**
		0.9	**2.7**	1.3	**3.8**	0.0	**2.5**
		128	128	128	128	32	64

Data is shown per row: 1^st^—outcome shown as their statistical / biological significance; 2^nd^—average log (CFU cm^-2^) reduction achieved compared to the positive control; 3^rd^—CST + AMP concentrations (the same concentration was used for both combined AMP). The more positive outcomes (S, Ad and ≥ 2 log reduction) are shown in bold.

Overall, the more positive outcomes (synergy and additiveness) were obtained with CST and TP-I-L combination for the reference and CI double-species biofilms. The CI MDR + CI MRSA double-specie biofilm appeared to be more susceptible to the combinations, achieving positive outcomes for all three, which further demonstrates the potential of AMP combinations in treating resistant infections. The 6 h treatment time appears to be slightly better for the reference and CI MDR + CI MRSA double-species biofilms. Also, the outcomes were different depending on the solid media used to assess biofilm-cell viability, which can be due to the different quantities of each bacteria in the 24 h-old biofilm. In fact, although the initial bacterial cell number was the same, the 24 h-old double-species biofilms were predominantly composed of *P*. *aeruginosa* (data not shown). Another interesting observation is that the AMP concentrations required to achieve positive outcomes in the double-species biofilms is superior to those needed for single-species biofilms. Finally, and as in the previous cases, statistical analysis of the biofilm biomass data deemed all combination outcomes as indifferent.

### Live/Dead inspection

**Fig 5:**
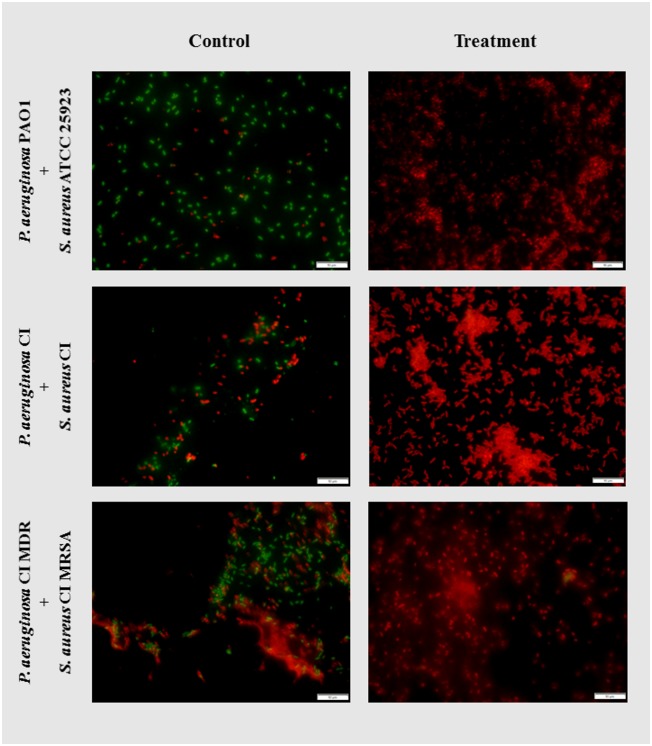
Live/Dead microscopy images of double-species biofilms treated with AMP combinations. The treatments applied were: CST + TP-I-L (128 mg L^-1^) for the biofilms of *P*. *aeruginosa* PAO1 + *S*. *aureus* ATCC 25923 and *P*. *aeruginosa* CI + *S*. *aureus* CI, and CST + CIT-1.1 (128 mg L^-1^) for the biofilms of *P*. *aeruginosa* CI MDR + *S*. *aureus* CI MRSA.

The effectiveness of the best AMP combination treatment towards double-species biofilms was evaluated qualitatively by analysing cells vitality through the Live/Dead fluorescence microscopy. In Fig 5, it is possible to observe that the amount of red-stained (dead) cells was relatively higher than in the controls. In all the three treatments, no green-stained (alive) cells were observed. Interestingly, the relative amount of adhered cells were generally higher in the treated coupons in comparison with the controls. This might indicate that the AMP combination treatment causes structural changes in the biofilm, namely enforcing cell adhesion and/or matrix production, which could be a defence mechanism/stress response of the bacteria. Yet, this hypothesis has to be further investigated. Despite the larger amount of cells, the AMP combinations were able to permeate the bacterial membranes and cause cell dead as seen by the red fluorescing cells in Fig 5. This observation is in concordance with the CFU data, in which some experiments were capable of total or almost total biofilm eradication (data not shown).

### Cytotoxicity of the AMP on mammalian cells

**Fig 6:**
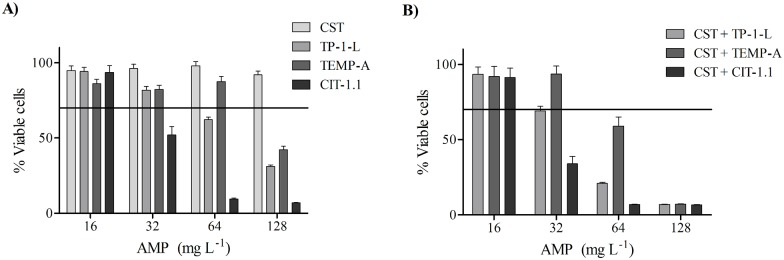
Fibroblast viability after 24 h of contact with the AMP alone (A) and combined (B). Data is depicted as bars representing the mean and error bars representing the standard error of the mean (SEM). Horizontal line indicates 70% of cell viability. Concentrations values were the same for each AMP.

As illustrated in Fig 6, results show that both individual and combined AMP were non-toxic (cell viability above 70%) [23] in concentrations ≤ 16 mg L^-1^. The least toxic AMP for fibroblasts was CST, showing no loss in viability up to 128 mg L^-1^. TEMP-A was non-toxic up to 64 mg L^-1^ and TP-I-L up to 32 mg L^-1^. CIT-1.1 was toxic at concentrations above 16 mg L^-1^. While AMP combinations were effective in terms of antimicrobial action, they did not exert synergy concerning their toxicity. As seen in Fig 6, AMP combinations were toxic at concentrations of ≥ 32 mg L^-1^, with the exception of CST + TEMP-A for 32 mg L^-1^. Presently, toxicity is shown for some of the effective combinations towards 24 h-old double-species biofilms.

## Discussion

In summary, this work has shown that combinations of CST with the AMP TEMP-A, CIT-1.1 and TP-I-L have synergic or additive actions in the prevention of planktonic growth, biofilm formation and treatment of pre-established single and double-species biofilms of *P*. *aeruginosa* and *S*. *aureus*. Individually, CST had higher effectiveness against *P*. *aeruginosa* in detriment of *S*. *aureus*, which was expected since CST acts by permeating the bacterial outer membrane by interacting with the anionic LPS molecules [24,25]. However, combinations of CST with the other AMP gave rise to synergic and additive outcomes, even for *S*. *aureus*, for both planktonic and biofilm growth. Since no anti-adhesive properties have been described for these peptides, one of the probable effects observed is the bactericidal effect of the AMP, which impaired biofilm development from the beginning.

All four AMP have their mechanism of action described as targeting the bacterial membrane [26–29], but TP-I also acts inside the cell by binding to DNA [30]. CST is mainly active against Gram-negative bacteria, TP-I and CIT-1.1 are considered wide-spectrum and TEMP-A is mainly active against Gram-positive bacteria. The reasons behind the positive interactions of CST with the other AMP are still elusive, but the synergy effects obtained are indicative of cooperation and demonstrates the capacity of synergic combinations to enlarge the antimicrobial spectrum of the combined agents. Mechanistically, it is plausible that positive outcomes occur in several ways due to the diversity of mechanisms of action found in AMP [31]. For example, one AMP can reach a threshold concentration more quickly than the other, facilitating the action of the second AMP inside the cell (no need to reach threshold concentration in this case), such as TP-I. Also, if the two AMP can act at the intracellular level, the synergic outcome could be the result of a multi-hit process for specific targets within the cell, but this is probably not the case here.

Concerning the 24 h-old biofilms, their eradication posed further challenges for AMP given the higher concentrations used compared to the prevention of biofilm formation. Biofilm has several resistance mechanisms, including the presence of an extra polymeric matrix that contains antibiotic-binding polymers and antibiotic-degrading enzymes that can impair AMP from reaching the cells [8]. In the case of the 24 h-old double-species biofilms, *P*. *aeruginosa* was the most prevalent of the two bacteria, which was expected, since *P*. *aeruginosa* and *S*. *aureus* are known to have a competitive relation, with *P*. *aeruginosa* disturbing *S*. *aureus* growth [6,32]. The concentrations of the AMP combinations required to achieve positive outcomes were higher in comparison with the single-species biofilms. This can be related to the fact that *P*. *aeruginosa* triggers the appearance of small colony variants (SCV) in *S*. *aureus*, which are more resistant to antibiotics and AMP [6]. Also, *P*. *aeruginosa* selectively lysis *S*. *aureus* by producing the enzyme LasA and it has been shown that the iron-containing proteins released from the lysed *S*. *aureus* cells can serve as an iron source for *P*. *aeruginosa*, increasing its pathogenic potential [33].

Overall, different times (2 h and 6 h) and media (PBS and TSB) of antimicrobial treatment did not have a clear influence on AMP activity against single-species biofilms. A longer treatment time (6 h vs 2 h) appears to slightly improve AMP activity against double-species biofilms, which may indicate a mixed time- and concentration-dependent killing for this scenario. The toxicity of the AMP combinations in mammalian cells did not demonstrate synergic outcomes and higher concentrations needed for double-species biofilm treatment demonstrated toxicity. Despite, the less effective but non-toxic concentrations could be of use when taking into account the combined action of these AMP with the host immune system.

The hereby tested AMP combinations were effective against reference and CI strains, as well as achieved synergic outcomes against biofilms of MDR and MRSA strains, including double-species biofilms. These findings pointed out the potential of combining AMP with established antibiotics (CST) for increasing treatment efficacy for biofilm-associated infections. CST showed to be non-toxic in all the range of concentrations tested, which emphasises its potential to be used in antimicrobial combinations. In order to overcome the issue of cytotoxicity of the AMP, several strategies are being addressed: the use of a matrix disrupting enzymes, such as dispersin B and alginate lysase, in order to allow AMP to more readily reach the biofilm cells; treatment regimens encompassing consecutive AMP doses in order to achieve biofilm eradication with lower AMP concentrations; finally, immobilization of the AMP, which has been proven to lower cytotoxicity [34]. Also, the study of the underlying mechanisms of these AMP interactions would be relevant to further infer about resistance evolution control and to strengthen the use of combinatorial AMP approaches.

## Supporting information

S1 Fig Overview of the AMP combination results for the inhibition of *P*. *aeruginosa* biofilm growth. Outcomes are shown as their statistical / biological significance. The more positive outcomes (S and Ad) are shown in bold.
(TIF)

S2 Fig Overview of the AMP combination results for the inhibition of *S*. *aureus* biofilm growth. Outcomes are shown as their statistical / biological significance. The more positive outcomes (S and Ad) are shown in bold. The outcomes marked with an asterisk were assessed in only one experiment.
(TIF)

S3 Fig Overview of the AMP combination results for the treatment of *P*. *aeruginosa* PAO1 and *S*. *aureus* ATCC 25923 double-species biofilm. Outcomes are shown as their statistical / biological significance. The more positive outcomes (S and Ad) are shown in bold.
(TIF)

S4 Fig Overview of the AMP combination results for the treatment of *P*. *aeruginosa* CI and *S*. *aureus* CI double-species biofilm. Outcomes are shown as their statistical / biological significance. The more positive outcomes (S and Ad) are shown in bold.
(TIF)

S5 Fig Overview of the AMP combination results for the treatment of *P*. *aeruginosa* CI MDR and *S*. *aureus* CI MRSA double-species biofilm. Outcomes are shown as their statistical / biological significance. The more positive outcomes (S and Ad) are shown in bold.
(TIF)

S1 File Heatmaps for the checkerboard data of *P*. *aeruginosa*. The FICI / FBCI values are shown for the combinations demonstrating an inhibition ≥ 99%.
(PDF)

S2 File Heatmaps for the checkerboard data of *S*. *aureus*. The FICI / FBCI values are shown for the combinations demonstrating an inhibition ≥ 99%.
(PDF)
