# Supplementary figures and images for: Searching for new strategies against biofilm infections: Colistin-AMP combinations against Pseudomonas aeruginosa and Staphylococcus aureus single- and double-species biofilms
Source: PLoS One. 2017 Mar 29;12(3):e0174654. doi: 10.1371/journal.pone.0174654 (PMC5371341; doi:10.1371/journal.pone.0174654)

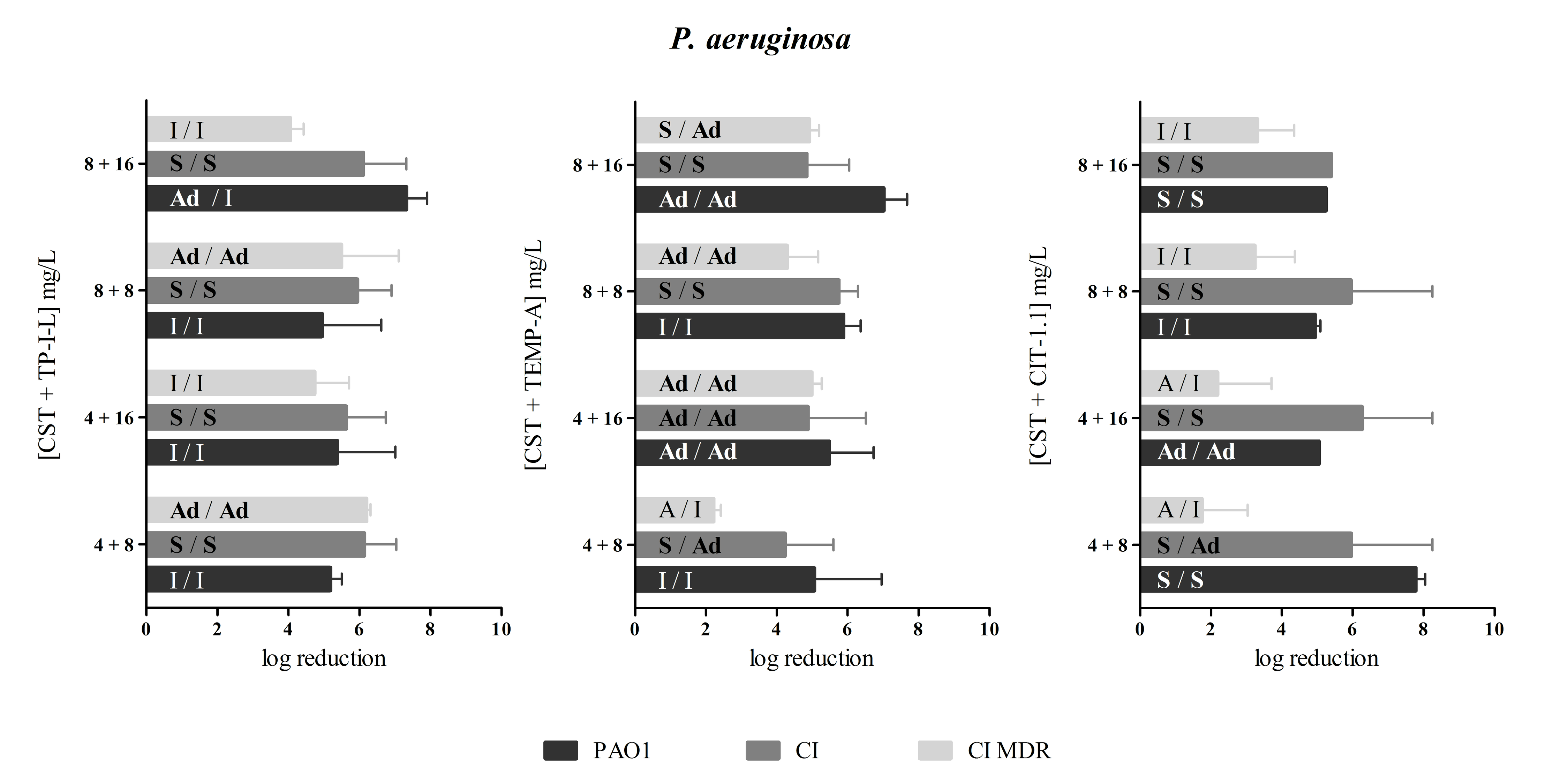

Supplement: S1 Fig — Outcomes are shown as their statistical / biological significance. The more positive outcomes (S and Ad) are shown in bold. (TIF) [file pone.0174654.s001.tif]

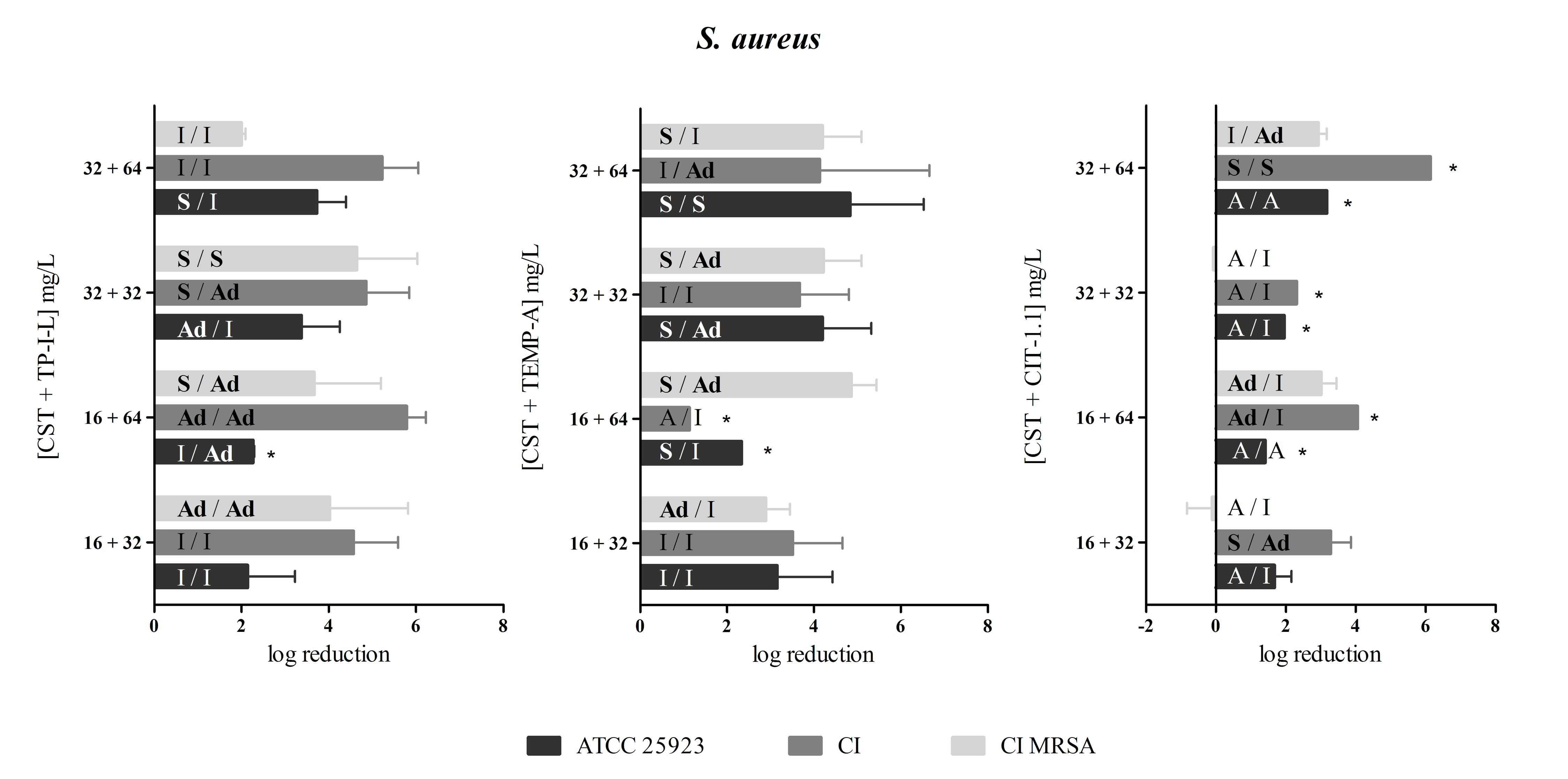

Supplement: S2 Fig — Outcomes are shown as their statistical / biological significance. The more positive outcomes (S and Ad) are shown in bold. The outcomes marked with an asterisk were assessed in only one experiment. (TIF) [file pone.0174654.s002.tif]

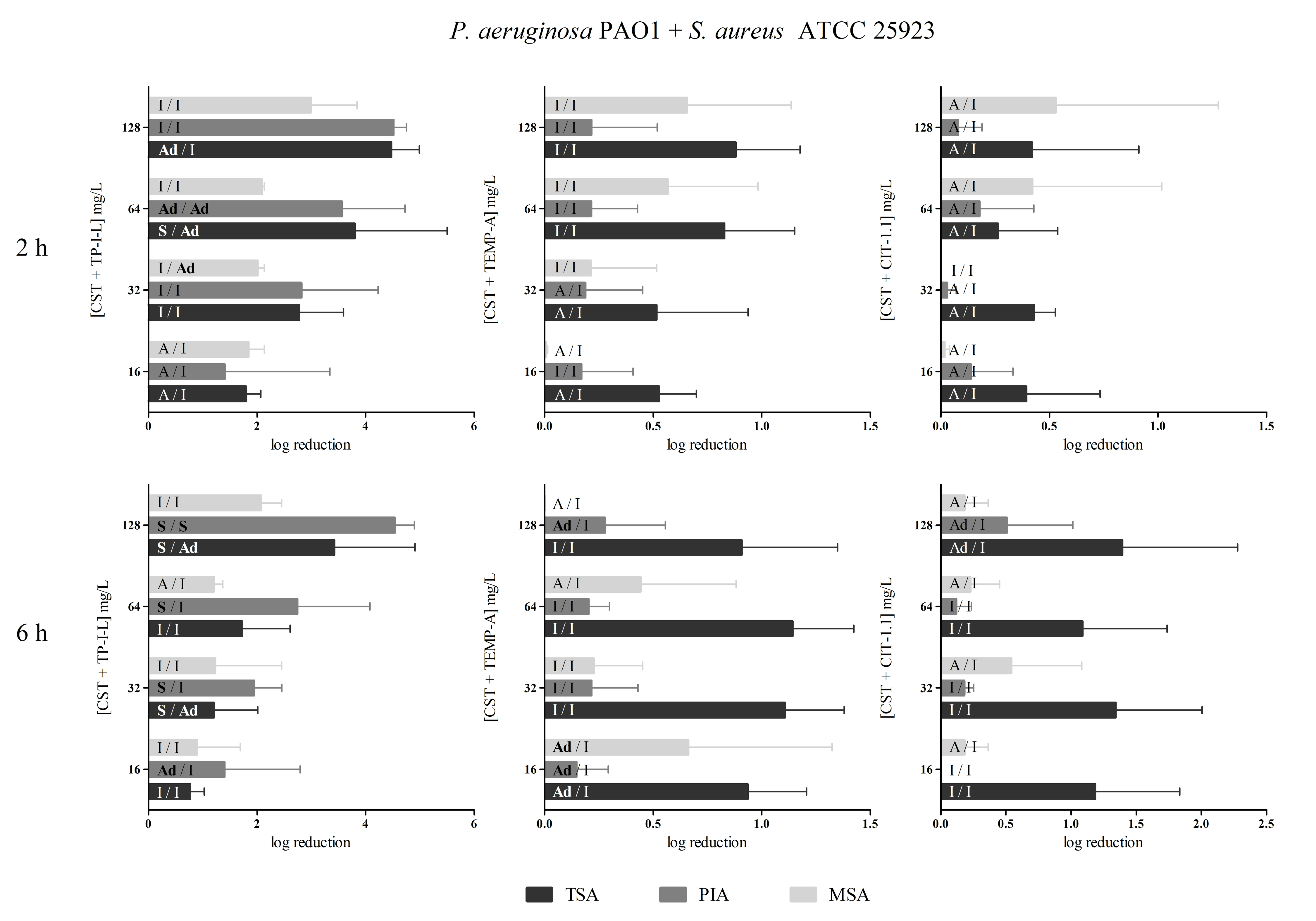

Supplement: S3 Fig — Outcomes are shown as their statistical / biological significance. The more positive outcomes (S and Ad) are shown in bold. (TIF) [file pone.0174654.s003.tif]

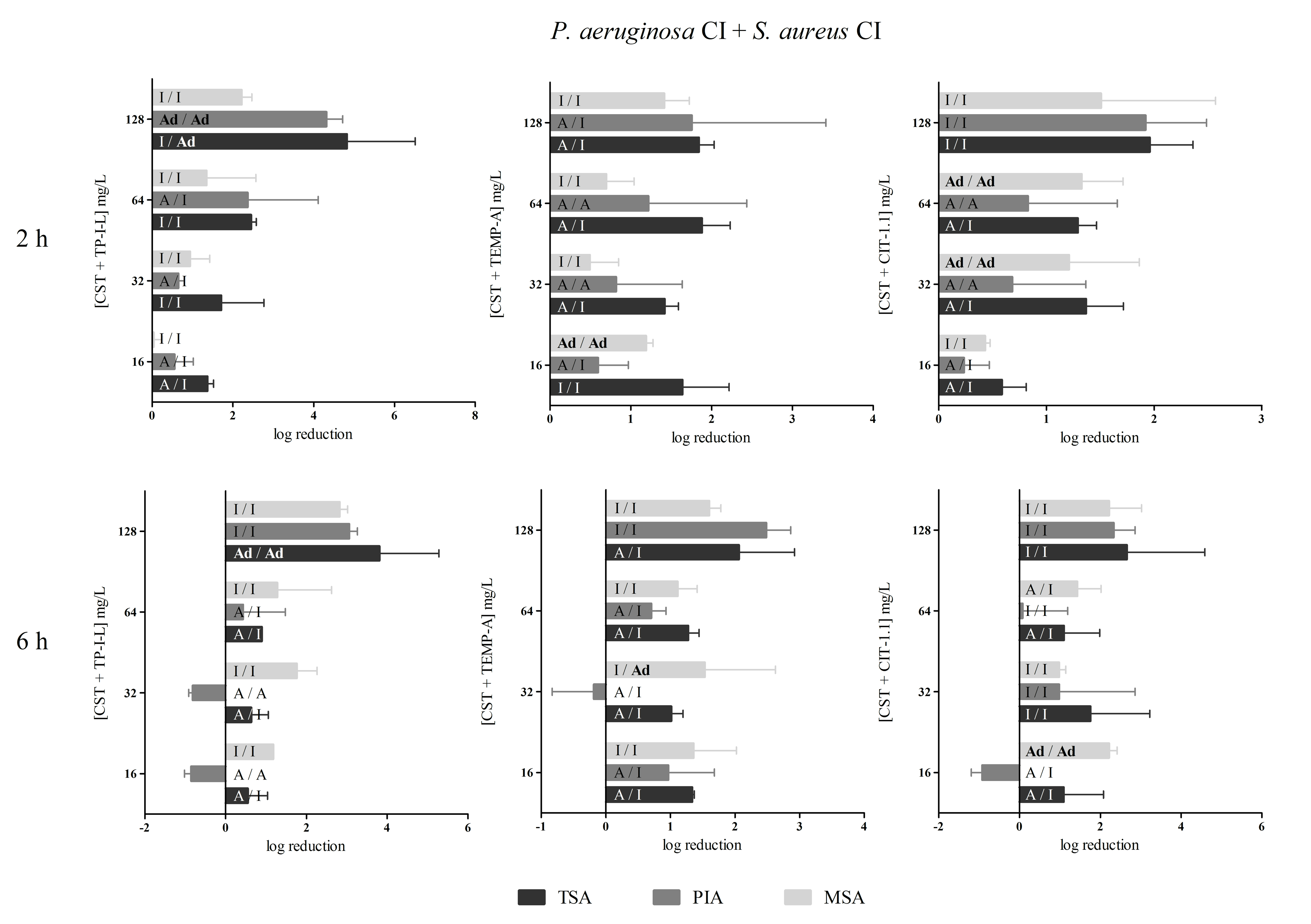

Supplement: S4 Fig — Outcomes are shown as their statistical / biological significance. The more positive outcomes (S and Ad) are shown in bold. (TIF) [file pone.0174654.s004.tif]

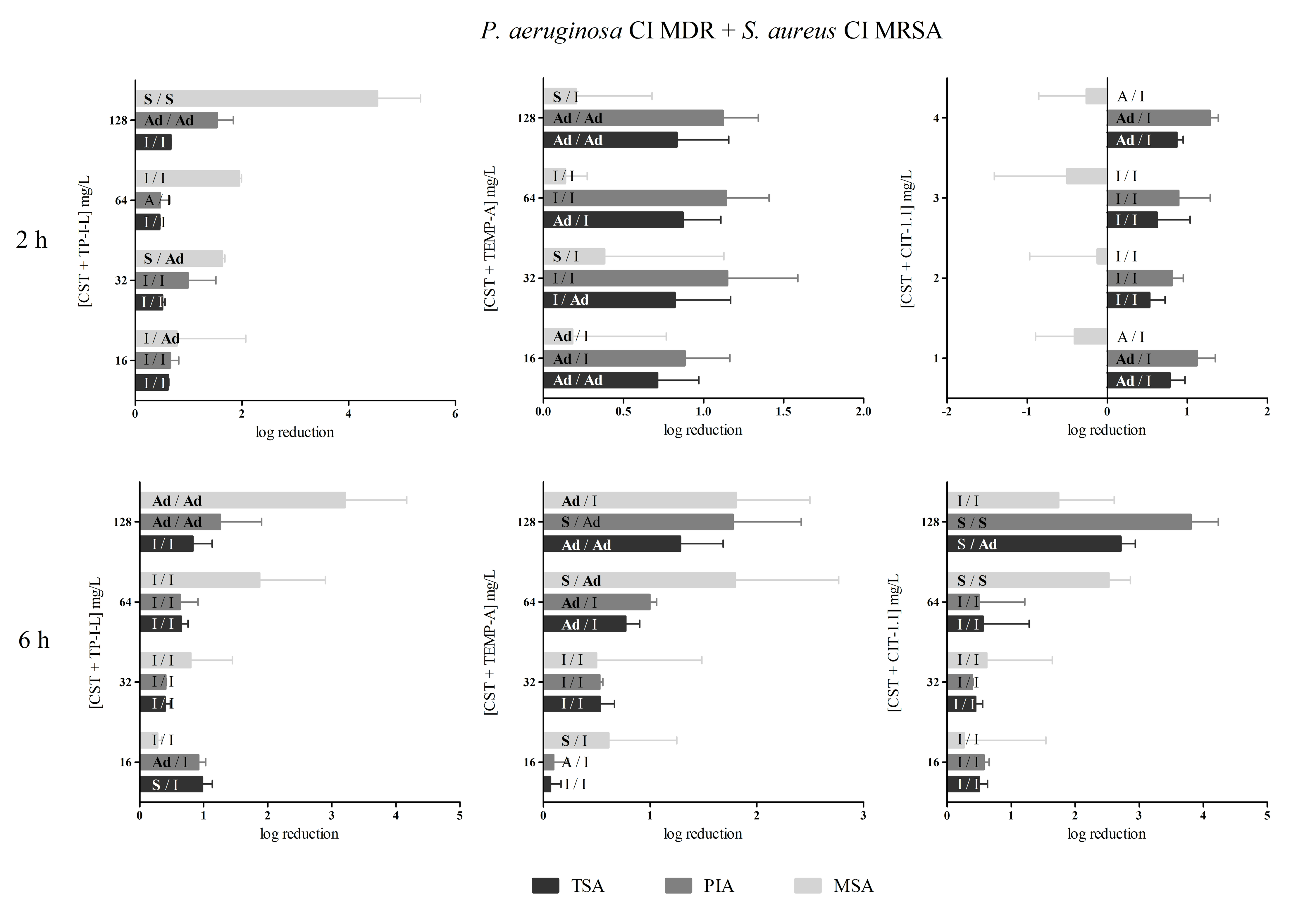

Supplement: S5 Fig — Outcomes are shown as their statistical / biological significance. The more positive outcomes (S and Ad) are shown in bold. (TIF) [file pone.0174654.s005.tif]

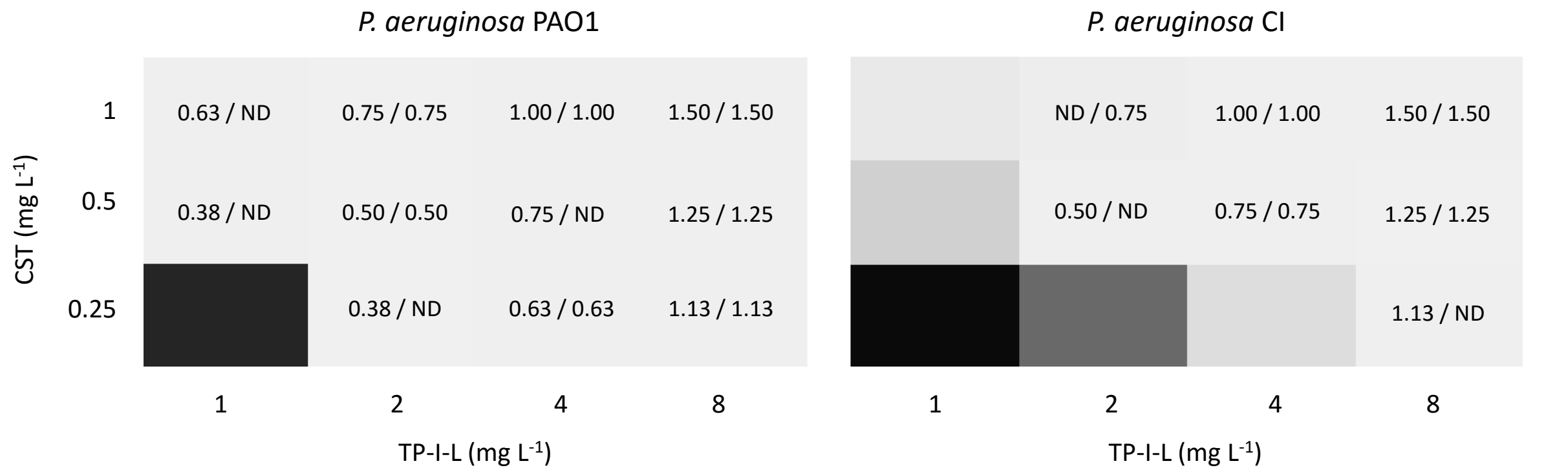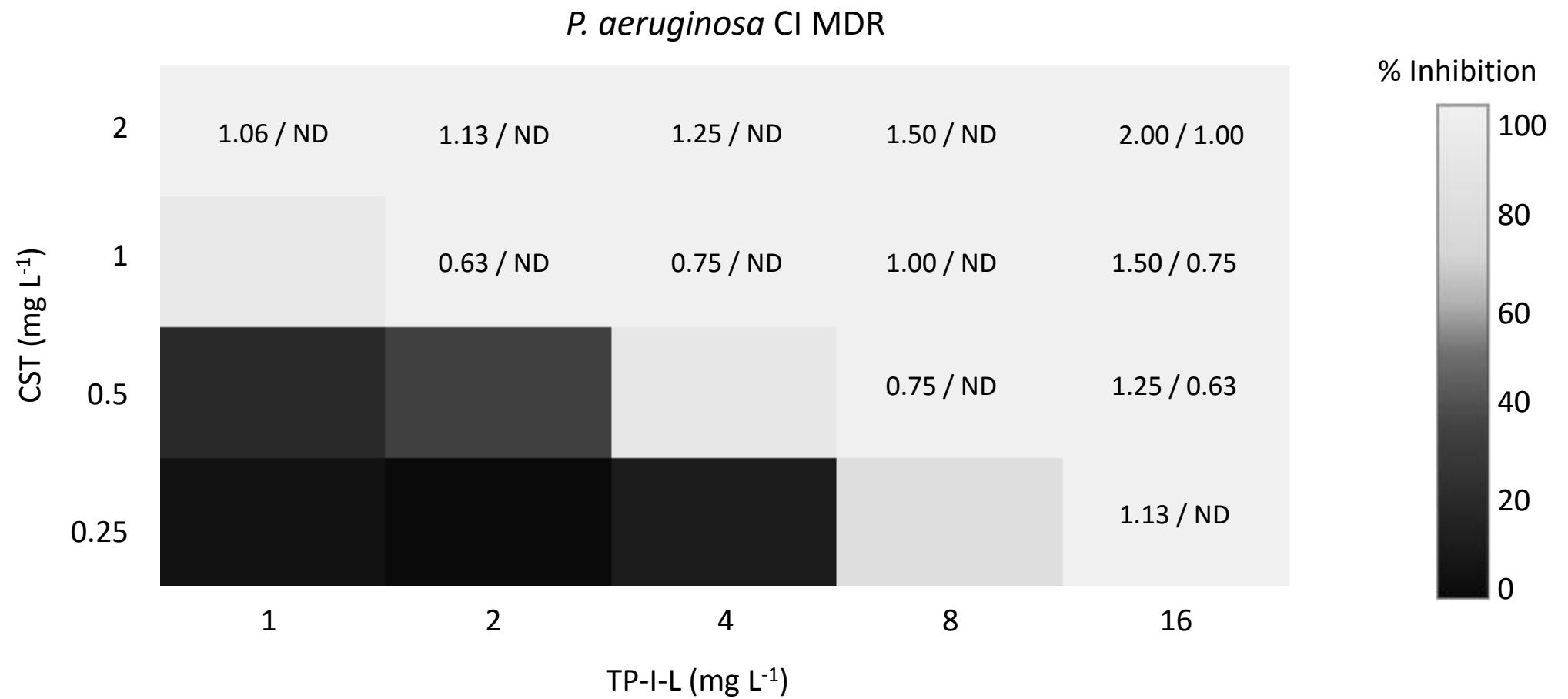



*P. aeruginosa* PAO1

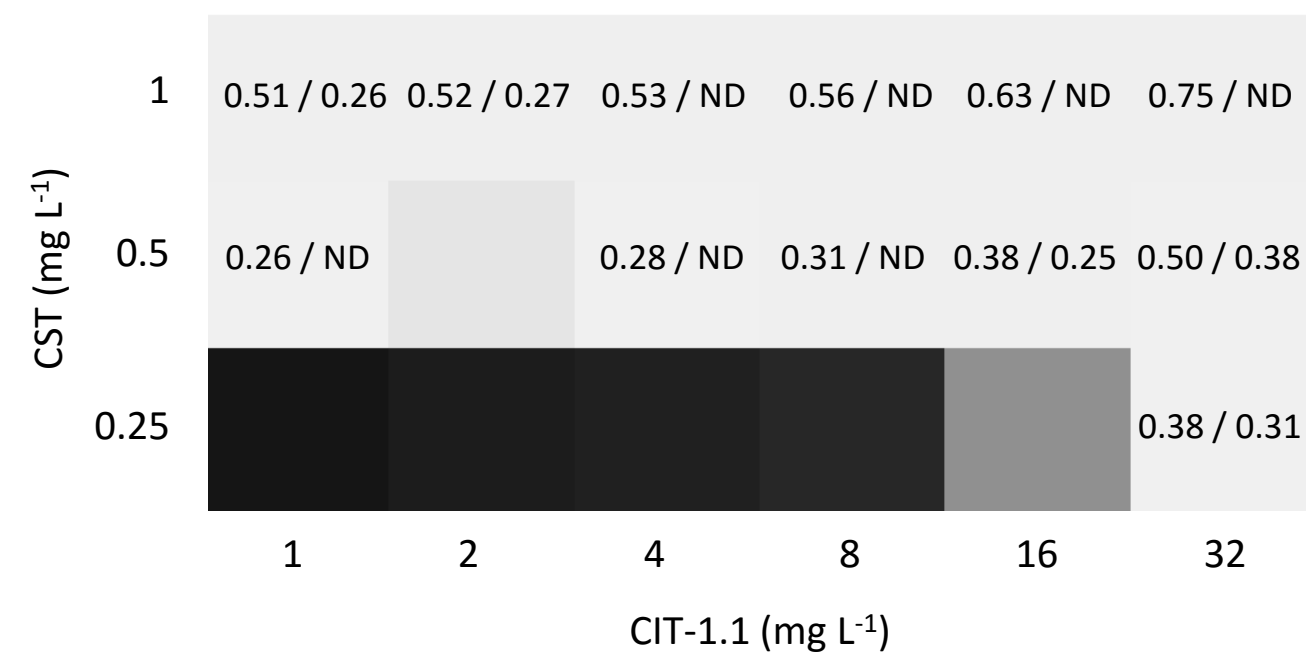

*P. aeruginosa* CI

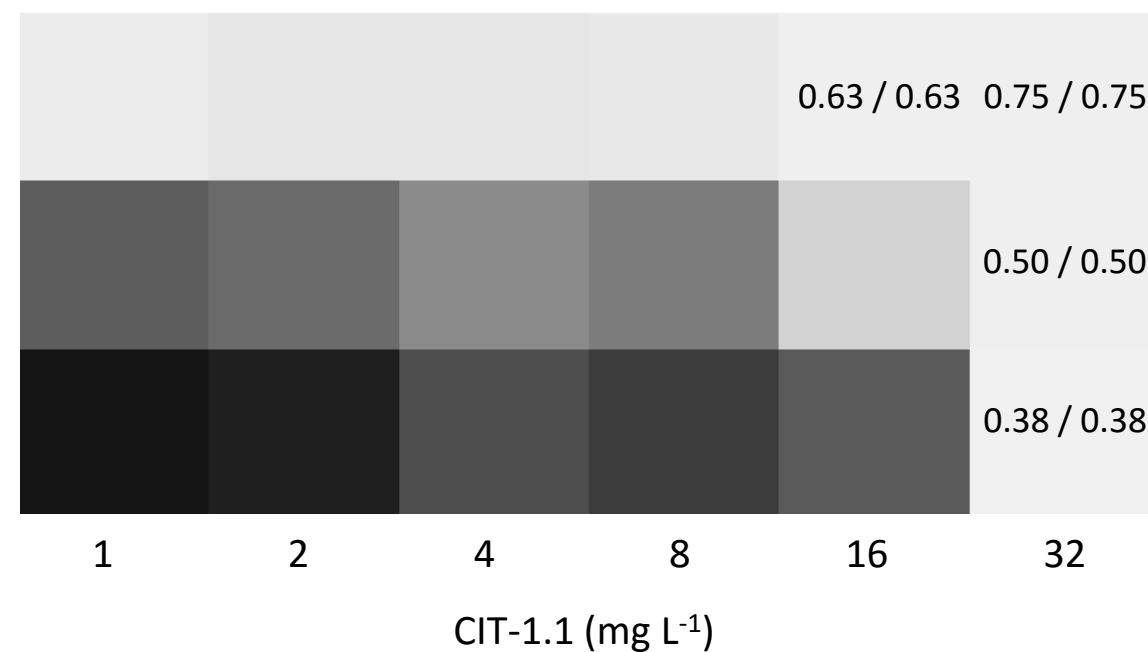

*P. aeruginosa* CI MDR

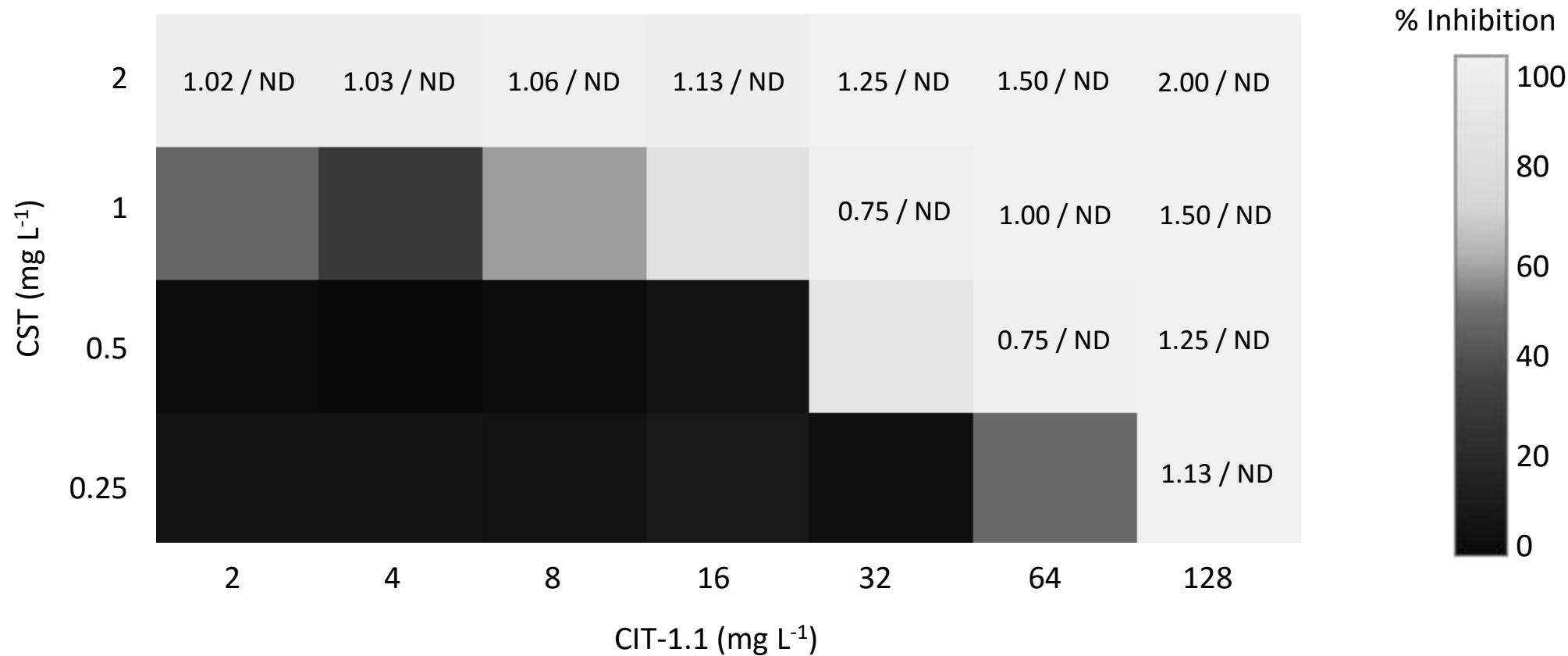

Supplement: S1 File — The FICI / FBCI values are shown for the combinations demonstrating an inhibition ≥ 99%. (PDF) [file pone.0174654.s006.pdf]

*S. aureus* ATCC 25923

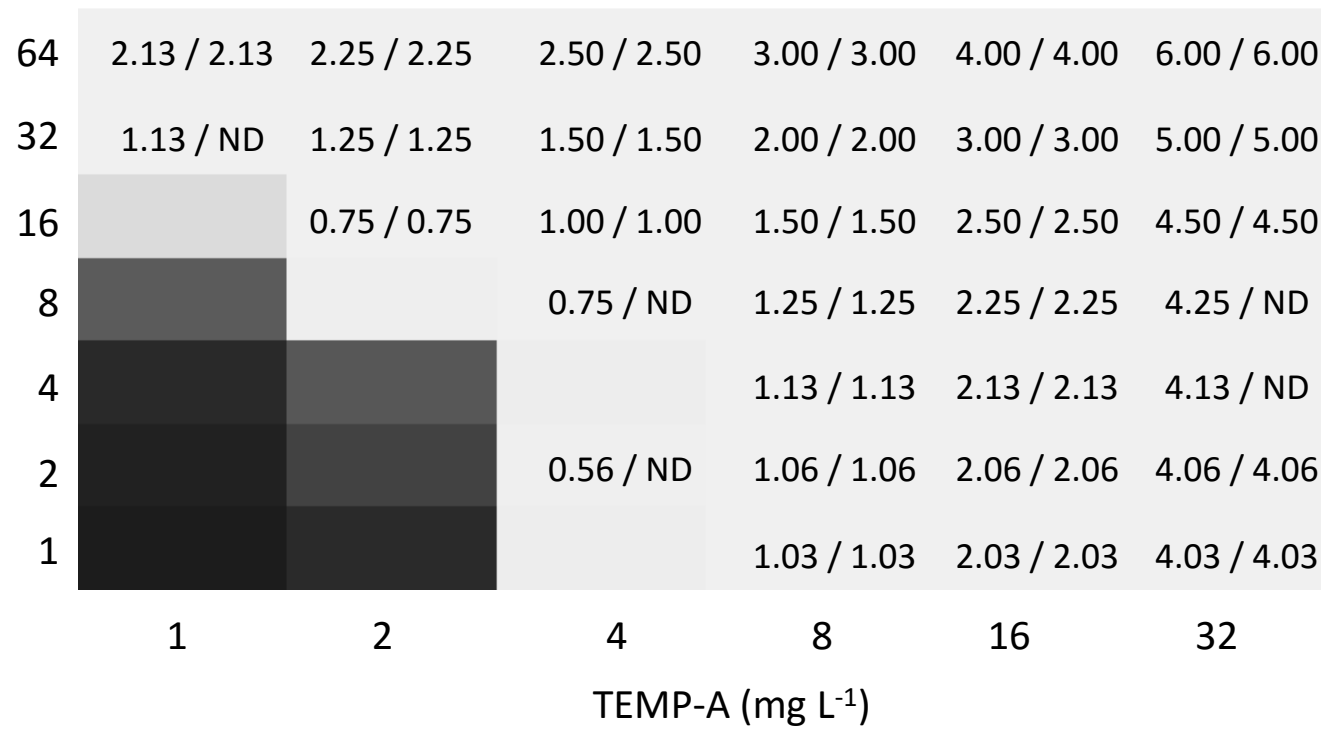

*S. aureus* CI

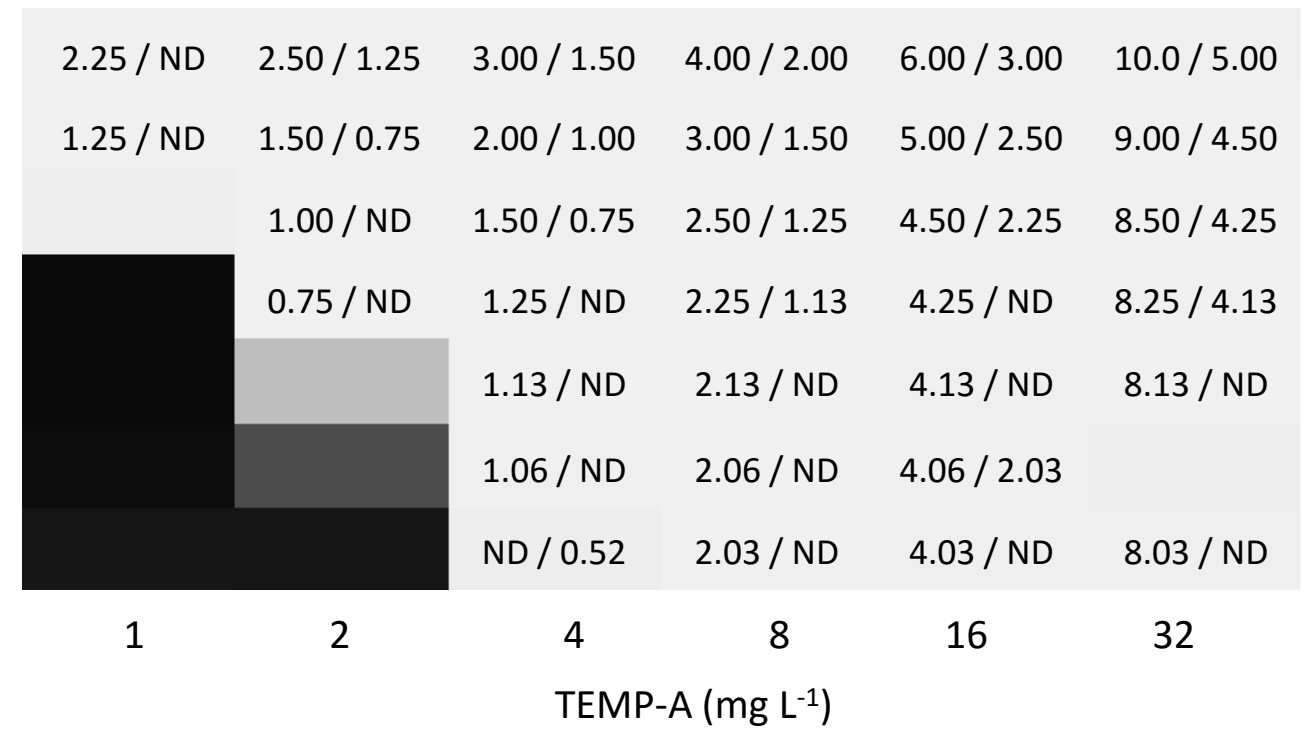

*S. aureus* CI MRSA

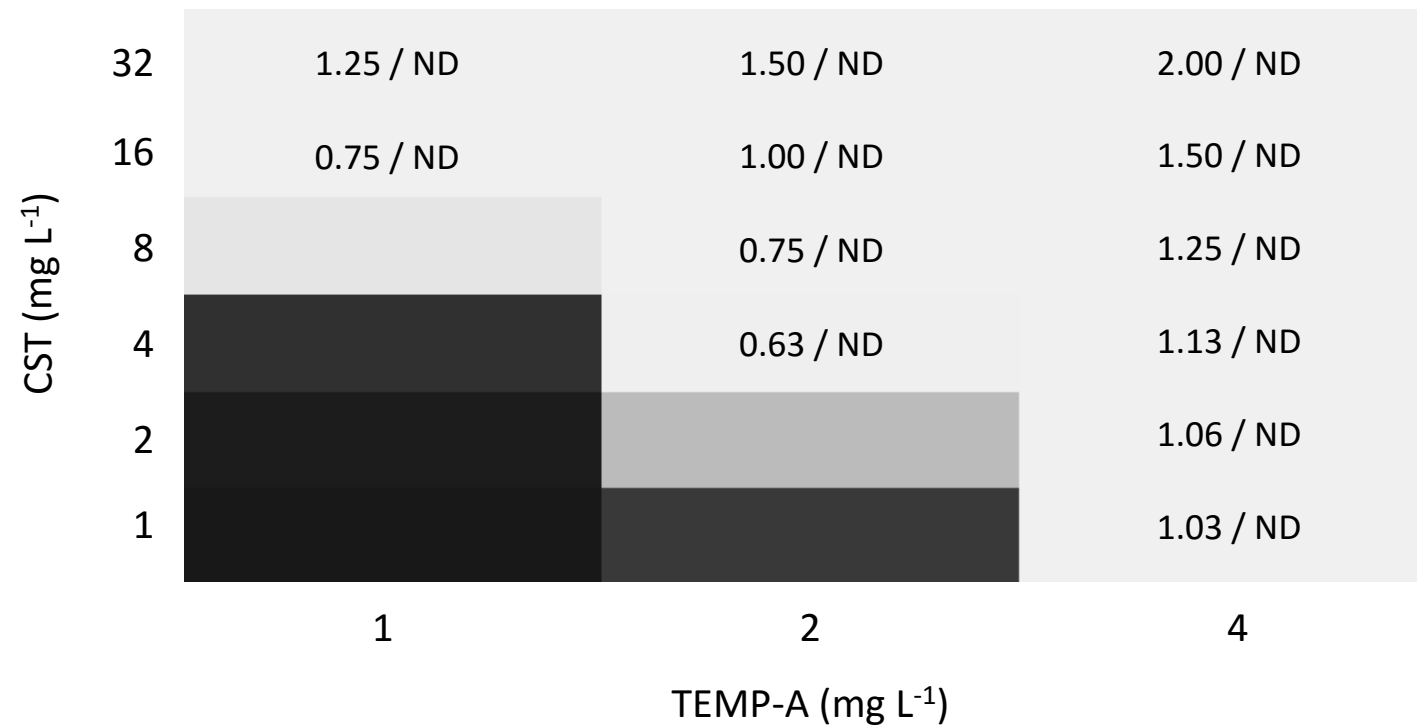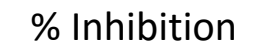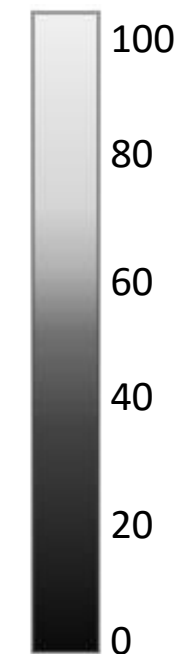

*S. aureus* ATCC 25923

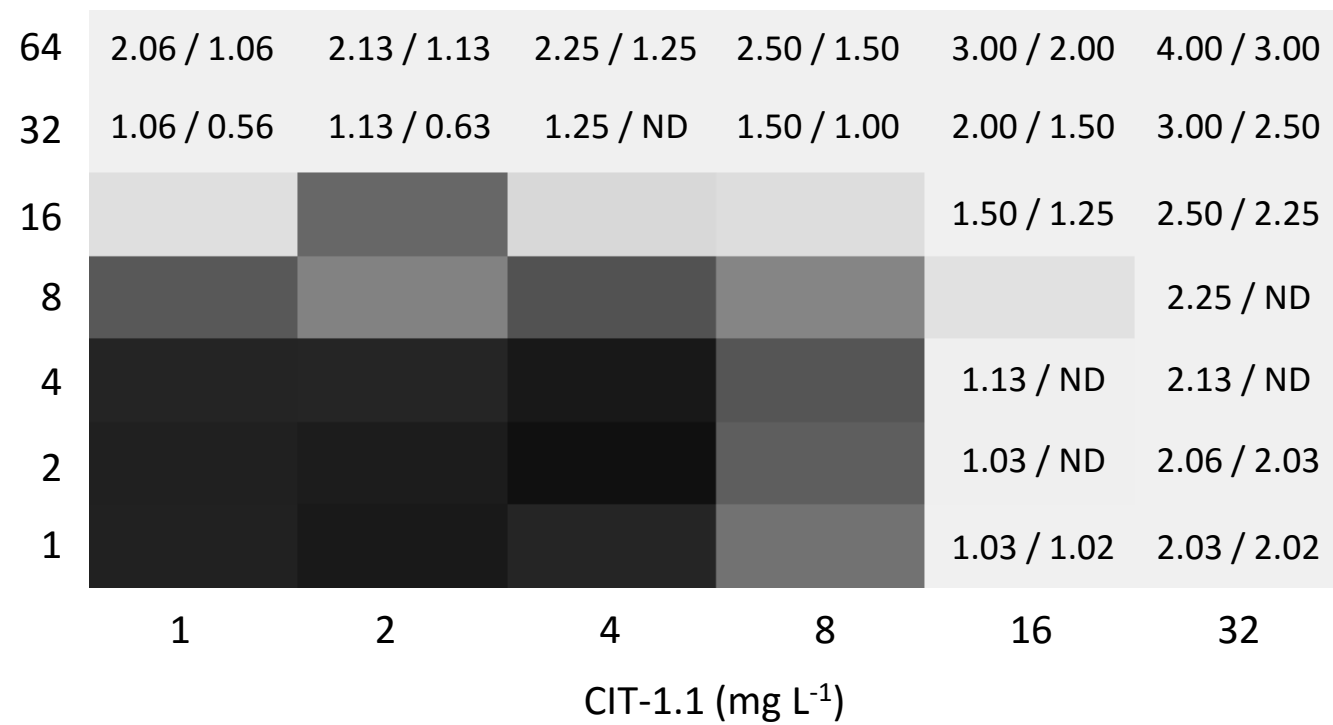

*S. aureus* CI

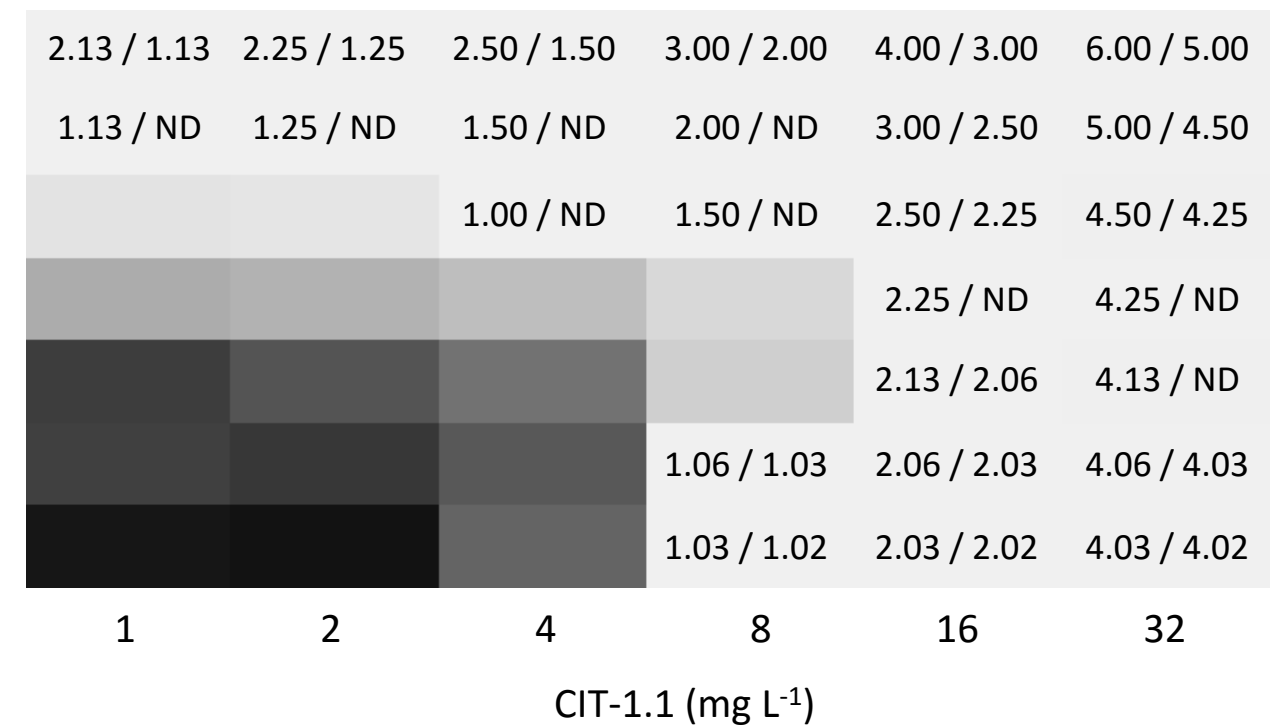

*S. aureus* CI MRSA

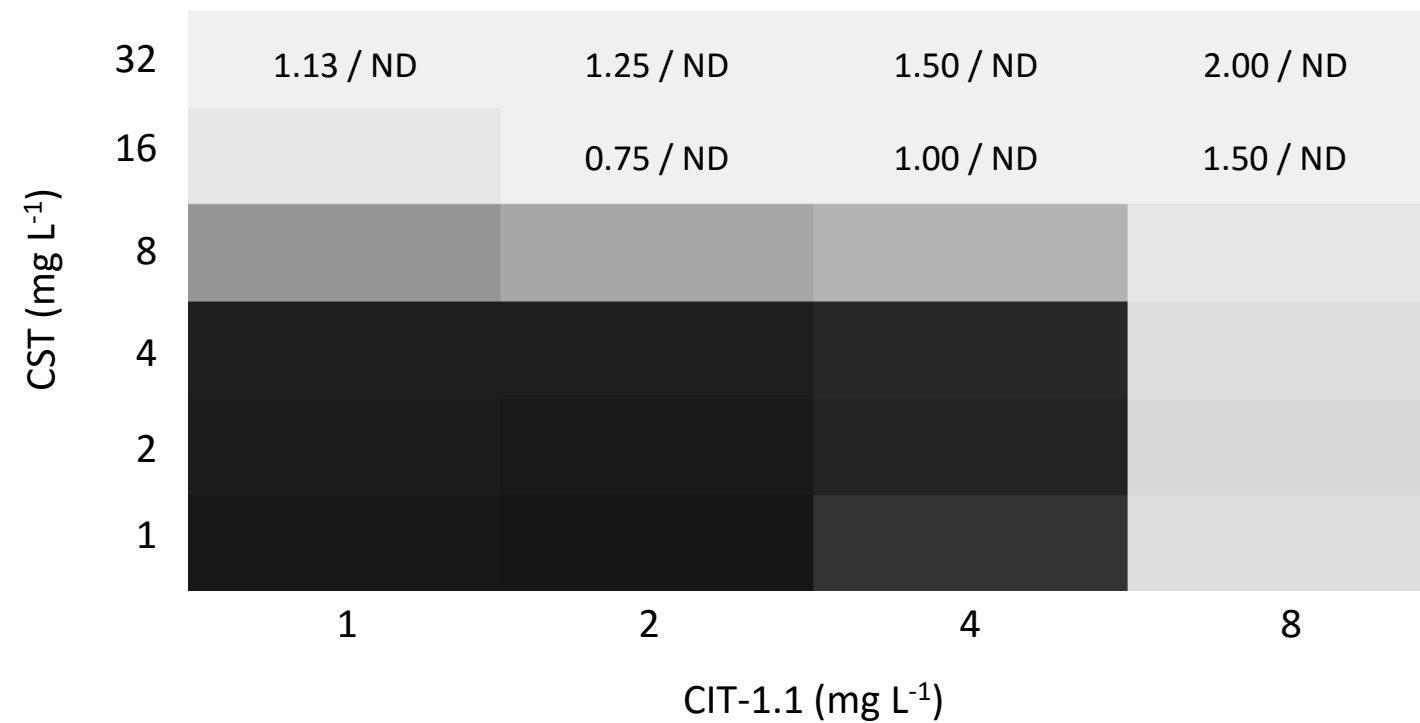

% Inhibition

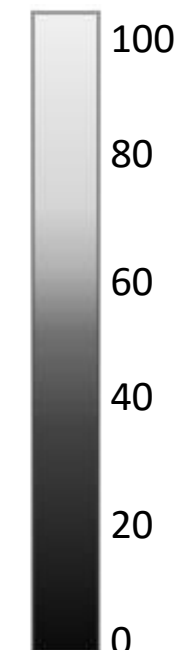

Supplement: S2 File — The FICI / FBCI values are shown for the combinations demonstrating an inhibition ≥ 99%. (PDF) [file pone.0174654.s007.pdf]
